# SNP Discrimination by Tolane-Modified Peptide Nucleic Acids: Application for the Detection of Drug Resistance in Pathogens

**DOI:** 10.3390/molecules25040769

**Published:** 2020-02-11

**Authors:** Kenji Takagi, Tenko Hayashi, Shinjiro Sawada, Miku Okazaki, Sakiko Hori, Katsuya Ogata, Nobuo Kato, Yasuhito Ebara, Kunihiro Kaihatsu

**Affiliations:** 1 Department of Organic Fine Chemicals, The Institute of Scientific and Industrial Research, Osaka University, 8-1 Mihogaoka, Ibaraki, Osaka 567-0047, Japan; takagi33@sanken.osaka-u.ac.jp (K.T.); haya33@sanken.osaka-u.ac.jp (T.H.); sinistergale@yahoo.co.jp (S.S.); okazaki33@sanken.osaka-u.ac.jp (M.O.); hori_s@visgene.com (S.H.); ogata-k@visgene.com (K.O.); kato-n@sanken.oska-u.ac.jp (N.K.); 2 Graduate School of Human Development and Environment, Kobe University, 3-11 Tsurukabuto, Kobe, Hyogo 657-8501, Japan

**Keywords:** peptide nucleic acid, tolane, single nucleotide polymorphism, influenza virus, drug resistance

## Abstract

During the treatment of viral or bacterial infections, it is important to evaluate any resistance to the therapeutic agents used. An amino acid substitution arising from a single base mutation in a particular gene often causes drug resistance in pathogens. Therefore, molecular tools that discriminate a single base mismatch in the target sequence are required for achieving therapeutic success. Here, we synthesized peptide nucleic acids (PNAs) derivatized with tolane via an amide linkage at the N-terminus and succeeded in improving the sequence specificity, even with a mismatched base pair located near the terminal region of the duplex. We assessed the sequence specificities of the tolane-PNAs for single-strand DNA and RNA by UV-melting temperature analysis, thermodynamic analysis, an in silico conformational search, and a gel mobility shift assay. As a result, all of the PNA-tolane derivatives stabilized duplex formation to the matched target sequence without inducing mismatch target binding. Among the different PNA-tolane derivatives, PNA that was modified with a naphthyl-type tolane could efficiently discriminate a mismatched base pair and be utilized for the detection of resistance to neuraminidase inhibitors of the influenza A/H1N1 virus. Therefore, our molecular tool can be used to discriminate single nucleotide polymorphisms that are related to drug resistance in pathogens.

## 1. Introduction

A single nucleotide polymorphism (SNP), as a variation at a single position in a gene sequence among individuals [1], within viral genes often confers drug resistance to the pathogen, such as HIV-1 drug resistance [2] or oseltamivir-resistant influenza virus [3]. Therefore, the sequence-specific detection of SNPs in target genes while using oligonucleotides is a key technology for detecting pathogens and disease-related genes. The accuracy and sensitivity of diagnosis relies on the chemical properties of the oligonucleotides that were used for detection. Thus, various types of chemically modified nucleic acids have been developed to improve the binding affinity and sequence specificity. Peptide nucleic acids (PNAs) are DNA mimics in which the phosphate backbone has been replaced by a neutral amide backbone composed of *N*-(2-aminoethyl)glycine linkages [4]. The advantages of PNAs are their high binding affinity [5,6,7], good mismatch discrimination [8], nuclease and protease resistance [9], and low affinity for proteins [10].

Up to now, PNAs have been used to detect single nucleotide polymorphisms (SNPs) through a combination with various types of other technologies. Ross et al. have utilized PNAs to detect SNPs in target genes while using matrix-assisted laser desorption/ionization time-of-flight mass spectrometry (MALDI-TOFMS) of PCR products. This ability to detect SNPs can be achieved, because PNAs can form stable complexes with their target genes under low salt conditions [11]. In order to perform the simultaneous detection of multiple SNP sites in dsDNA by MALDI-TOFMS, Ren et al. mixed multiple PNAs and dsDNA and treated them first with exonuclease III and then with nuclease S1 to produce PNA/ssDNA fragments, including the SNP sites in situ. They succeeded in discriminating between various apolipoprotein E genotypes in patients while using dsDNAs that were obtained by PCR [12]. Boontha et al. developed a new ion-exchange capture technique for SNP detection using a pyrrolidinyl PNA probe. The complementary PNA/DNA hybrid is selectively captured by the anion exchanger in the presence of noncomplementary or unhybridized PNA, allowing for the direct detection of the hybridization event on the anion exchanger by MALDI-TOFMS. The accuracy of MALDI-TOFMS, in conjunction with the high specificity of PNA hybridization, offers promise for development into a multiplexed, high-throughput screening technique [13].

Gaylord et al., developed a method for the fluorescence-based detection of SNPs while using PNA probes conjugated with an optically amplifying conjugated polymer poly[(9,9-bis(6′-*N*,*N*,*N*-trimethylammoniumhexylbromide) fluorene)-co-phenylene], and S1 nuclease. Recognition is accomplished by the sequence-specific hybridization between the uncharged, fluorescein-labeled, PNA probe, and the DNA sequence of interest. After subsequent treatment with S1 nuclease, the cationic polymer electrostatically only associates with the remaining anionic PNA/DNA duplex, leading to the sensitized fluorescence emission of the labeled PNA probe via FRET from the cationic polymer [14]. They succeeded in detecting a known point mutation that had been implicated in a dominant neurodegenerative dementia known as frontotemporal dementia with Parkinsonism linked to chromosome 17 (FTDP–17), which has clinical and molecular similarities to Alzheimer’s disease.

The fluorescence-based detection of SNPs is a simple, rapid, and robust technology. Rockenbauer et al. reported a new method that combined allele-specific hybridization, PNA technology, and detection while using flow cytometry. These authors described a fully functional two-bead genotyping system based on PNA capture and flow cytometric detection that they used for the accurate and fast re-genotyping of a Danish basal cell carcinoma cohort [15]. Bethge et al. synthesized forced intercalation probes (FIT-probes) that contained an intercalating cyanine dye, such as oxazole yellow (YO), which serves as a replacement for a canonical nucleobase. The YO in the FIT probes responds to adjacent base mismatches through the attenuation of fluorescence intensity under conditions where both matched and mismatched target DNAs are bound. The YO-PNA is capable of signaling the presence of fully complementary DNA by providing an up to 20-fold enhancement in fluorescence. Single base mismatches cause a significant attenuation of YO fluorescence [16]. Socher et al. also employed a thiazole orange (TO) modified FIT-PNA molecule to monitor SNPs in a target gene. They found that the use of D-ornithine rather than aminoethylglycine as the PNA backbone increased the intensity of the fluorescence emitted by matched probe-target duplexes, while the specificity of fluorescence under non-stringent conditions was also increased. The utility of these ornithine-containing FIT probes was demonstrated in a real-time PCR analysis providing a linear measurement range over at least seven orders of magnitude [17]. Ditmangklo et al. synthesized novel alkyne-modified styryl dyes for conjugation with pyrrolidinyl peptide nucleic acid (acpcPNA) while using click chemistry to detect the presence of structural defects, including mismatched, abasic, and base-inserted DNA targets. The largest increase in the fluorescence quantum yield (up to 14.5–fold) of styryl-dye-labelled acpcPNA was achieved with DNA carrying base insertions [18].

Kam et al. succeeded in discriminating SNPs in the KRAS oncogene in cultured cells while using two types of molecular beacons (MBs) based on either phosphothioated DNA (PS-DNA-MB) or peptide nucleic acid (TO-PNA-MB, where TO = thiazole orange). Cell transfection of TO-PNA-MB with the aid of PEI resulted in fluorescence in cells expressing the fully complementary RNA transcript (Panc-1), but undetectable fluorescence in cells expressing the K-ras mRNA that had a single mismatch to the TO-PNA-MB (HT29). In contrast, PS-DNA-MB showed no fluorescence in all the cell lines that were tested post PEI transfection [19]. Further, Kolevzon et al. synthesized a PNA bis-quinoline that was capable of detecting mutant K-ras mRNAs in cultured cells by monitoring far-red emission [20].

As mentioned above, PNA is a powerful tool that can be used to discriminate SNPs when used in combination with a mass spectrometer, PCR, or a fluorescence detector. The usefulness of PNA will be further amplified if it could be used for the rapid diagnosis of viral and bacterial drug-resistance in clinical specimens. This could be achieved by improving the hybridization properties of the PNA, eliminating the need for any expensive equipment. We recently reported that the hybridization property of a PNA could be enhanced by azobenzene modification at the *N*-terminus [21]. We also attached intercalators containing larger pi-conjugated systems, such as acridine and pyrene to the *N*-terminus of PNA via amide linkages. However, these modifications increased binding not only to the matched DNA, but also to the mismatched DNA (data not shown). Dogan et al. [22] reported that modification by stilbene, an orthogonal molecule, at the 5′-terminus of the DNA, also enhanced the binding affinity for matched DNA/DNA duplex formation, without increasing the formation of mismatched duplexes. These reports inspired us to explore modification of the *N*-terminus end of PNA with a more rigid and orthogonal molecule when compared to stilbene, which could increase the binding affinity and sequence specificity of PNA to DNA and RNA.

In this study, we designed and synthesized various types of intercalators utilizing a diphenylachetylene(tolane) backbone and attached them to the N-terminus of the PNA via amide linkage. The binding affinities and sequence specificities of these PNA derivatives for DNA or RNA were assessed by a UV-melting temperature analysis and a gel mobility shift assay. We also developed a novel type of rapid diagnostic test kit for discriminates SNP relating neuraminidase inhibitor-resistant virus of the seasonal influenza virus.

## 2. Results and Discussion

### 2.1. Design and Synthesis of PNA and Tolane-PNA

**Figure 1:**
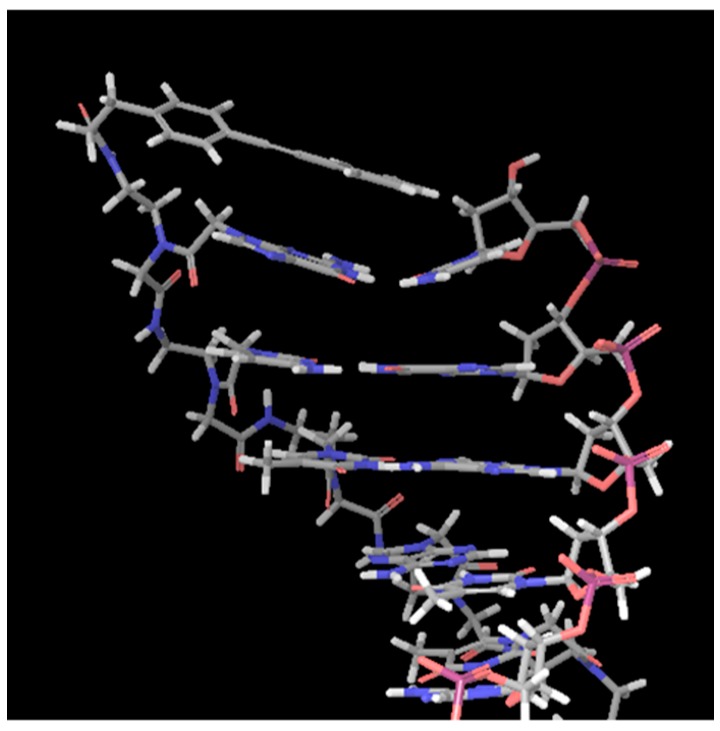
Conformational search of the tolane**1** modified Peptide nucleic acid (PNA)/DNA duplex. The original PNA/DNA duplex structure was obtained from the Protein Data Bank (PDB:1PDT). The conformational search was performed while using the force field OPLS2005 model in MacroModel.

**Figure 2:**
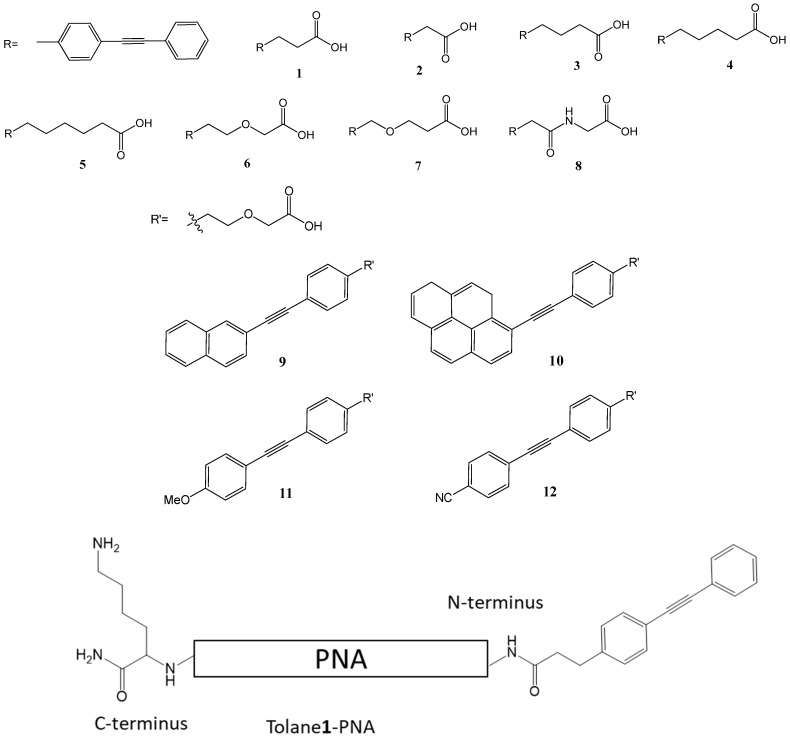
Chemical structure of the tolane derivatives. **1**–**5**) tolanes containing different alkyl linker lengths, **6–8**) tolanes containing either ether or amide linkages, **9**–**12**) tolanes containing different pi-conjugation systems. Tolane**1**-PNA) Schematic diagram of tolane**1** modified PNA. A lysine residue was introduced to the C-terminus of the PNA molecule to increase water solubility. Tolane**1** was attached to the N-terminal amino group through an amide linkage.

The typical duplex structure of PNA/DNA has been reported in the Protein Data Bank (PDB:1PDT). We introduced a diphenylacetylene (tolane) derivative to the *N*-terminal of PNA via an amide linkage and performed an in silico conformational search while using MacroModel to design a chemically modified PNA that can form a stable stacking conformation with the terminal base pairs (Figure 1). As can be seen in Figure 1, the tolane molecule (Figure 2, tolane**1**) appeared to form a stacking interaction with a neighboring base pair in the terminal region of the PNA/DNA duplex. We reasoned that the optimization of the linker length in tolane might further enhance the stacking conformation in the PNA/DNA duplex. Accordingly, we synthesized a series of tolane molecules that had different linker lengths or pi-conjugation systems (Figure 2). These were introduced to the N-terminal region of a 12-mer poly-pyrimidine PNA (PNA**0**) that is known to be sensitive to mismatches and it is unable to form a triplex with the target DNA (Table 1). The lysine at the C-terminus was introduced to increase the water solubility of the PNA.

### 2.2. The Effect of Linker Structures in the Tolane-PNAs on Duplex Stability with Single Strand DNA

The thermal stabilities of the tolane-PNA/DNA duplexes were used to assess the effects of the different tolane derivatives on duplex stability. We measured the melting temperature (Tm) of PNA**0–12** with a single stranded DNA (DNA**1**). The Tm of the matched PNA**0**/DNA**1** was 56.5 °C, while that of the mismatched PNA**0**/DNA**2** was 48.6 °C, thus representing a difference of 7.9 °C.

PNAs **1–5** represent molecules with different lengths of the alkyl chain spacer that lies between the PNA and the tolane moiety. The Tms of their duplexes with DNA**1** increased in an alkyl length-dependent manner until the tolane linker was pentanoic acid (Table 2. PNA**4**, 61.7 °C). The PNA**5**/DNA1 duplex, which contained a hexanoic acid linker, had a lower Tm than the PNA**4**/DNA**1** duplex (Table 2. 59.6 °C). Pentanoic acid seemed to be an optimal linker length for tolane-PNA conjugates based on these data. Interestingly, the Tms of the PNA**0-5**/DNA2 mismatched duplexes did not increase with alkyl chain length (Table 2. Tm = 48.1–48.8 °C). Among PNA**0–5**, PNA**4** gave the largest ΔTm (+13.0 °C), which was 5.1 °C larger than that of PNA**0** (ΔTm = +7.9 °C). Tolane**4** in PNA**4** could efficiently associate with adjacent base pairs in the matched PNA/DNA duplex and it also stabilized the duplex formation.

**Figure 3:**
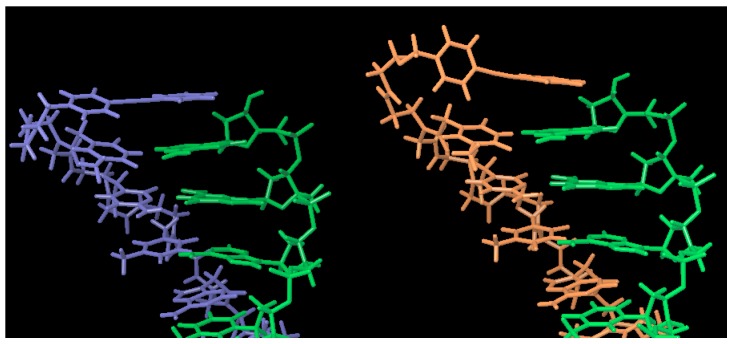
Molecular docking simulation of the tolane-PNA/DNA duplex conformation. Left: tolane**6** in PNA forms a favorable stacking conformation with the terminal base pair of the PNA/DNA duplex via its flexible ether linker. Right: tolane**8** in PNA forms a stacking interaction with the DNA bases, but not with the terminal base of the PNA due to the rigidity of the amide backbone. The PNA/DNA duplex structures were obtained as 1PDT from the PDB data bank and the conformational search was performed using the force field OPLS2005 model and MacroModel. Green; DNA, Blue; PNA conjugated with tolane**6**, Orange; PNA conjugated with tolane**8**.

We further investigated the effect of the chemical structure of the tolane linkers on PNA/DNA duplex formation. We elected to use a tolane linker length, which was the same as hexanoic acid; however, we substituted the aliphatic linkers with two more flexible and hydrophilic ether linkers (Figure 2, tolane**6–7**) or a more rigid amide linkage (Figure 2. tolane**8**). The two different types of ether linkers and the amide linker were introduced to the N-terminus of PNA, as shown in Table 1 (PNA**6–8**). PNA**6** and PNA**7** both contain flexible ether linkers that increased duplex stability with DNA**1** relative to PNA**4** (Table 2, PNA**6**/DNA**1** Tm = 63.0 °C, PNA**7**/DNA**1** Tm = 62.2 °C). In contrast, PNA**8** contains a more rigid amide linker that had a slightly lower Tm (Table 2, PNA**8**/DNA1 Tm = 60.9 °C) when compared to PNA**4**. We performed a conformational search of tolane**6** and tolane**8** in the PNA/DNA duplex (PDB:1PDT) by an in silico conformational search while using MacroModel^®^ to understand the mode of binding of tolane molecules within the PNA/DNA duplex. As a result, the flexible ether linker of tolane**6** efficiently formed stacking conformation with the terminal base pairs, while the rigid amide linked tolane**8** did not (Figure 3).

**Figure 4:**
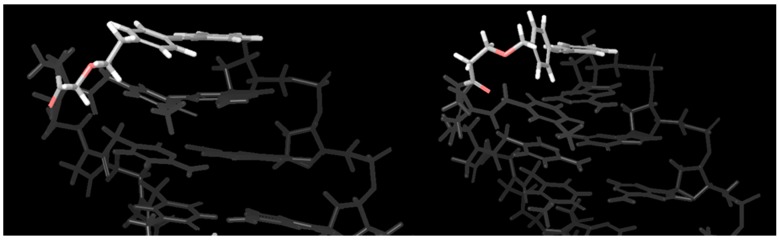
Molecular docking simulation of the tolane-PNA/DNA duplex conformation. Left: tolane**6** formed a favorable stacking conformation with the terminal base pair of the PNA/DNA duplex via its flexible ether linker. Right: tolane**7** was unable to form a stacking interaction with the N-terminal base pair due to the rigidity of the amide linker. The PNA/DNA duplex structures were obtained as 1PDT from the PDB data bank and the conformational search was performed with the OPLS2005 force field and gradient termination at 0.001 kJ/mol-Å (MacroModel, 2010).

We performed a conformational search using the same procedures to understand the different effects of tolane**6** and tolane**7**. As a result, tolane**6** formed a stable stacking interaction with the adjacent PNA/DNA base pair, while tolane**7** could only form a stacking interaction with the DNA bases (Figure 4). Regardless of the flexibility of the linker, PNA **6** and **7** did not show an increase in the Tm for the duplex with mismatched DNA**2** (Table 2). From these results, we chose the ethoxyacetate linker for the further optimization of the tolane molecules.

### 2.3. Modification of the Tolane Structure to Enhance the Stacking Interaction with the PNA/DNA Duplex

**Figure 5:**
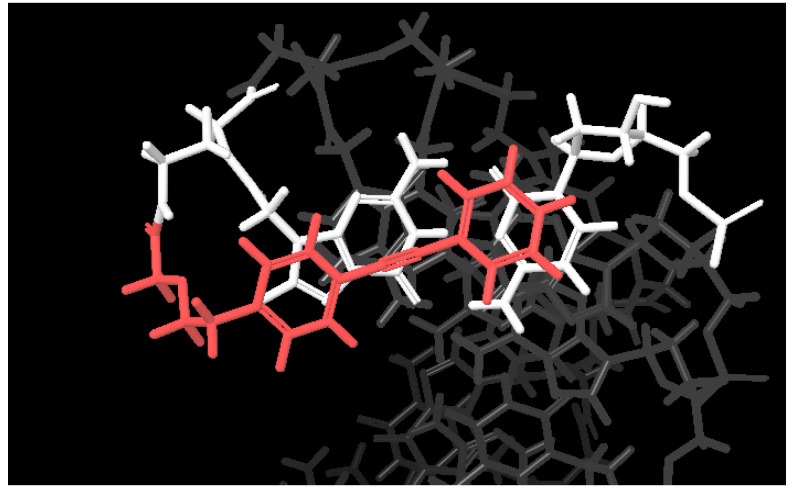
Conformational search of tolane6-PNA/DNA using MacroModel. Force Field; OPLS2005, Solvent: water, Red; tolane. White; terminal base pairs.

In the previous section, the optimization process showed that the ethoxyacetic acid linker (tolane**6**) was the best candidate among tolane**1–8**. We next modified the structure of the tolane backbone to enhance the stacking interaction with the terminal base pairs of the PNA/DNA. According to our in silico conformational search that is shown in Figure 5, an extended pi-conjugate system might increase the stacking interaction with the terminal base pair of PNA/DNA (Figure 5. Top view of the tolane-PNA/DNA duplex. Red: tolane**6**, White: terminal base pair).

Based on these data, we synthesized tolane**9** and **10**, which had extended pi-conjugated systems in their terminal phenyl group and tolane**11** and **12** that had an electron donor and an acceptor group, respectively. Each compound was attached to the amino group at the N-terminus of the PNA and the resulting DNA binding affinities were assessed by a UV-melting temperature analysis (Table 2). As a result, tolane**9**, which contains a naphthyl group, increased the duplex stability with DNA**1** (Table 2, Tm = 64.8 °C) and the thermal stability was found to be higher than that of tolane**6** (Tm = 63.0 °C). On the other hand, tolane**9** had almost no effect on the thermal stability of the mismatched PNA**9**/DNA**2** duplex (Table 2, Tm = 48.3 °C). As PNA **10** possesses a pyrene group, it has poor solubility in aqueous solution. Therefore, we used 20% methanol in a phosphate buffered solution to measure the Tm of DNA**1**. As a result, the pyrene containing tolane **10,** which has an enhanced pi-conjugated system and had a lower Tm when compared to tolane **9** (Table 2, Tm = 60.7 °C). Although the pi-conjugated system in tolane **10** is larger than that tolane **9**, the poor water solubility of tolane**10** made it unsuitable for forming a stacking interaction with the neighboring base pairs in the PNA **10**/DNA**1** duplex when in an aqueous solution.

We next examined the effect of functional groups at the para-position in tolane on the thermal stability of the PNA/DNA duplex. PNA**11,** which has an electron donating methoxy group in tolane**11,** had a lower Tm (Table 2, Tm = 60.9 °C) when compared to PNA**7**. The structural bulkiness of the methoxy group in tolane**11** might cause steric hindrance with the neighboring base pairs in PNA**12**/DNA**1** and prevent the stacking interaction. PNA**13**, which has an electron withdrawing cyano group in tolane**12,** had nearly the same Tm (Table 2, Tm = 63.3 °C) as PNA**6**. These data indicate that the electron density of the tolane molecule does not affect the stacking interaction with the terminal base pair of the tolane-PNA/DNA. Overall, the tolane derivatives did not stabilize the mismatch duplex formation of the tolane-PNA/DNA, regardless of the modified functional groups. Based on the ΔTm (16.5 °C) in Table 2, PNA**9,** which is a naphthyl type tolane (tolane **9**), had the best sequence specificity.

### 2.4. Analysis of the Duplex Conformation of the Tolane-PNA/DNA Duplexes by Fluorescence Spectroscopy

**Figure 6:**
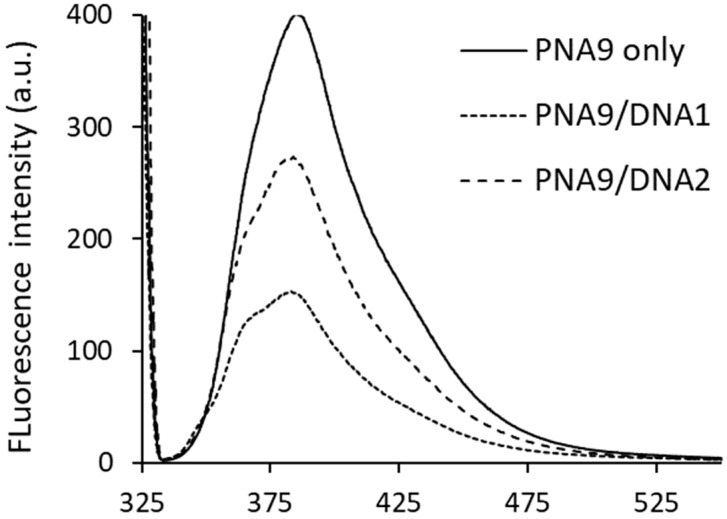
The fluorescence spectra of PNA**9** (solid), PNA**9**/DNA**1** (dashed-dotted line), and PNA**9**/DNA**2** (dashed line) were measured in 20 mM sodium phosphate buffer (pH 7.4). Conditions: PNA, DNA = 4 μM, Excitation = 323 nm, Emission = 384 nm, Temperature = 25 °C.

We analyzed the fluorescence spectra of PNA**9** in the absence or presence of matched DNA**1** or mismatched DNA**2** to understand the mode of action of tolane**9** in the PNA/DNA duplex. PNA**9** was dissolved in 20 mM phosphate buffer (pH 7.4) at a concentration of 4 μM at 25 °C. The excitation wavelength of tolane**9** in PNA**9** was found to be 323 nm, while the emission wavelength was found to be 384 nm (Figure 6, fluorescence intensity: 402.24). The fluorescence intensity was drastically quenched to 151.94 when PNA**9** was mixed with matched DNA**1** at a 1:1 molar ratio (Figure 6). This is likely to be as the result of the stacking interaction of tolane**9** with the neighboring base pairs in PNA**9**/DNA**1**. On the other hand, when PNA**9** was mixed with mismatched DNA**2** at a 1:1 molar ratio, the fluorescence intensity was decreased by about half relative to DNA**1** (Figure 6, fluorescence intensity: 271.33). These results indicated that tolane**9** could form an efficient stable stacking conformation with matched DNA**1**. When a penultimate base pair of the PNA/DNA was mismatched, tolane**9** did not form a stable stacking conformation with an adjacent base pair due to the fraying of the base pairs in the terminal region.

### 2.5. Thermodynamic Study of the PNA/DNA and Tolane-PNA/DNA Duplexes

**Figure 7:**
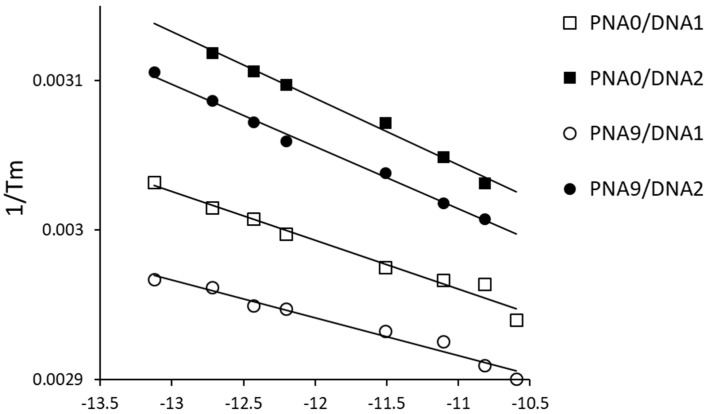
Tm plot of the PNA/DNA and tolane-PNA/DNA duplexes at different concentrations in 20 mM phosphate buffer (pH 7.4). The R/∆H values were calculated from the slope of each approximate straight line. The ∆S/∆H values were calculated from the estimated intercept on the 1/Tm line as ln(Ct/4) approached zero.

We analyzed the thermodynamic parameters of PNA**0** and PNA**9** with matched DNA**1** and mismatched DNA**2** to study the binding behavior of tolane**9**. The Tm values of the duplex varied depending on their total concentration. Thus, we measured the Tm of each duplex and plotted 1/Tm on the vertical axis and ln(Ct/4) on the horizontal axis, as shown in Figure 7. The enthalpy (∆H) was calculated from the slope of the approximate line obtained, and the entropy (∆S) was calculated from the intercept, as indicated in Equation (1) below. Figure 7 shows the actual plots and approximate straight lines. ∆G was calculated based on Equation (2).

1/Tm = (R/ΔH)ln(Ct/4) + ΔS/ΔH(1)

ΔG = ΔH − TΔS(2)

We obtained the R/ΔH for PNA**0**/DNA**1**, PNA**0**/DNA**2**, PNA**9**/DNA**1**, and PNA**9**/DNA2, which was 3.25 × 10^−5^, 4.47 × 10^−5^, 2.52 × 10^−5^, and 4.18 × 10^−5^ (K^−1^), respectively, according to the approximate straight line in Figure 7. In addition, the ΔS/ΔH ratio for PNA**0**/DNA**1**, PNA**0**/DNA**2**, PNA**9**/DNA**1**, and PNA**9**/DNA2 was calculated as 2.60 × 10^−6^, 2.55 × 10^−6^, 2.64 × 10^−6^, and 2.55 × 10^−6^ (K^−1^), respectively. The heat capacity should be negligible. The gas constant (R) was 0.00199 (kcal/(mol·K)). These data were used for calculating the ΔH, ∆S, and ΔG values, according to Equations (1) and (2), and they are summarized in Table 3.

The ΔG values in Table 3 showed good correlations with the Tm values. When comparing the ΔH of the matched PNA/DNA duplex, PNA**9** (−79.1 kcal/mol) had a smaller value than PNA**0** (−61.8 kcal/mol), which indicates that tolane**9** forms a stacking interaction with the neighboring base pairs by intermolecular bindings, such as pi–pi stacking interactions and hydrogen bonds. When comparing the ∆S of the matched PNA/DNA, PNA**9** (−208.8 cal/mol·K) had a smaller value than PNA**0** (−159.5 cal/mol·K), which indicates that the stacking interaction of tolane**9** impairs the structural flexibility at the terminal region of the PNA/DNA duplex. However, the enthalpy contribution was relatively bigger than the loss of entropy, so, as a result, the free energy of PNA**9** (−16.9 kcal/mol) was lower than PNA**0** (−13.7 kcal/mol) and formed a stable duplex. Looking at the thermodynamic parameters of the mismatched duplexes in Table 3 revealed that PNA**0** and PNA**9** had almost the same values. This suggested that tolane**9** has negligible interaction with PNA/DNA duplex when the mismatched base is located at the second base position from the terminus. This sequence specificity was caused by the structural rigidity of the tolane, since the diphenylacetylene backbone can adopt a stacking conformation only when the terminal base pair forms a matched duplex.

### 2.6. Recognition of Single Base Mismatched DNA by Tolane 9

**Figure 8:**
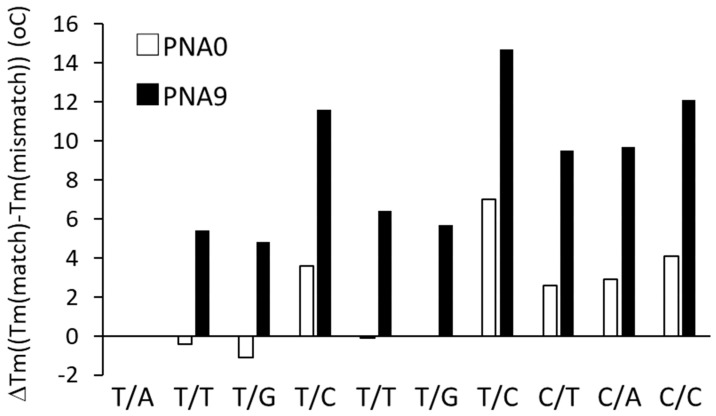
Measurement of Tm for PNA**0**/DNAs and PNA**9**/DNAs in 20 mM phosphate buffer (pH 7.4). ∆Tm = Tm (matched) − Tm (mismatched). DNA**1** contains a complementary sequence to the PNAs. DNA**3**, DNA**4**, and DNA**5** contain a mismatched base at the first, second, and third base from the N-terminus of the PNA.

We prepared DNA**3–5**, which had three single base mismatches at the first, second, and third bases from the 3′-terminus, respectively, to confirm that tolane**9** can discriminate a single base mismatch in its target DNA (Table 4). As summarized in Table 5, tolane**9** in PNA**9** slightly increased the Tm for the mismatched DNAs (DNA3: +0.3–+2.5 °C, DNA4: +0.8–2.6 °C, DNA5: +0.3–1.5 °C) compared to PNA**0**/DNA**3**–**4**. On the other hand, PNA**9** increased the Tm for the matched DNA by approximately 8.3 °C. Therefore, its sequence specificity is improved, as shown in Figure 8, as ∆Tm [∆Tm = Tm (matched) − Tm (mismatched), maximum ∆Tm; +14.7 °C (T/C mismatch with DNA**4**), minimum ∆Tm; +4.8 °C (T/G mismatch with DNA**3**)]. Although there are many other possible mismatch variations in the PNA/DNA duplex, we think that tolane**9** can improve the sequence specificity, regardless of the exact location of the mismatch.

### 2.7. Detection of Single Nucleotide Polymorphism (SNP) in a Neuraminidase Inhibitor-Resistant Influenza Virus by PNA**13** and PNA**14** Using a Gel Mobility Shift Assay

Neuraminidase inhibitors, such as Zanamivir and Oseltamivir, are antiviral medicines that are used to treat and prevent influenza virus infections. A SNP in the influenza A virus neuraminidase gene often causes drug-resistance to those neuraminidase inhibitors. Kawakami et al. identified the difference between a neuraminidase inhibitor-resistant influenza A viral gene (Yokohama/77/2008/H1N1, GenBank Accession number: AB465325) and a neuraminidase inhibitor-sensitive influenza A viral gene (Yokohama/1/2008/H1N1, GenBank Accession ID: AB519808) by direct sequencing of the PCR product of the neuraminidase gene during the previous swine influenza pandemic in 2008–2009 [23]. They found that nucleotide 823 (labeled from the 5′-terminus of the complementary viral RNA gene ((+) stand RNA), which causes a change from an adenine to a thymine, leads to the substitution of histidine to a tyrosine at amino acid 275 (H275Y). We synthesized PNA**13** and PNA**14** that are complementary to the viral gene in Influenza A/Yokohama/77/H1N1 to discriminate this SNP within the viral RNA((−) strand RNA). We attached a Lys-O-O-Lys-biotin linker (O; aminoethylethoxyacetate) to increase the water solubility and develop a nucleic acid chromatography method for the detection of a SNP associated with the neuraminidase inhibitor-resistance of influenza A/Yokohama/77/H1N1.

We first prepared DNAs that contained the sequences of influenza A/Yokohama/1/2008/H1N1 (Table 6, DNA**6**) and influenza A/Yokohama/77/2008/H1N1 (Table 6, DNA**7**) genes and performed a UV-melting temperature analysis. As a result, PNA**14** had a Tm that was 5.8 °C higher with the matched DNA**7** (Tm = 59.3 °C) compared to PNA**13**/DNA**7** (Tm = 53.5 °C). PNA**14** had nearly the same Tm with mismatched DNA**6** (47.6°C) relative to PNA**13**/DNA**6** (Tm 48.0 °C). As a result, the modification of tolane**9** to PNA enhanced the ∆Tm by 6.2 °C (i.e., from 5.5 °C (PNA**13**) to 11.7 °C (PNA**14**)). These results indicated that tolane**9** only increases the binding affinity to the target sequence when the sequence is matched.

We also assessed the binding of PNA**13** and PNA**14** to RNA sequences that were derived from influenza A/Yokohama/1/H1N1 (Table 6, RNA**1**) and influenza A/Yokohama/77/H1N1 (Table 6, RNA**2**) neuraminidase genes. Each RNA consisted of 17 bases and the 5′-terminus was fluorescently labelled with Cy5 to allow for the visualization of the binding on a gel (Table 6, RNA**1** and RNA**2**). PNA**14** showed a higher Tm with the matched RNA**2** (Table 7, Tm = 55.3 °C) when compared to PNA**13**/RNA**2** (Table 7, Tm = 51.7 °C). On the other hand, PNA**14** showed a significantly lower Tm with the mismatched RNA**1** (Table 7, Tm = 31.5 °C) as compared to PNA**13**/RNA**1** (Table 7, Tm = 40.2 °C). As a result, tolane**9** improved the ∆Tm of PNA by 23.8 °C (Table 7), which is 12.3 °C higher than PNA**13** (Table 7, ∆Tm = 11.5). We hypothesized that the lowered binding affinity of PNA**14** to the mismatched RNA could be due to the hydrophobic property of tolane**9**, which prefers to form intramolecular interactions with neighboring PNA bases and avoids forming a mismatched duplex with the hydrophilic RNA that possesses 2′-hydroxyl groups. While using a conformational search that was conducted using MacroModel, as shown in Figure S1 (supplemental data), we compared the stability of the capped state of tolane-PNA/DNA, in which tolane forms a stacking interaction with neighboring base pair in the PNA/DNA duplex, and the backbone binding state of the tolane-PNA/DNA duplex, in which tolane is bound to the PNA backbone. The capped state is 2 kcal more stable than the backbone binding state based on the ∆G energy. The difference in ∆G energy indicates that the relative abundance ratio of the capped state and backbone binding state within tolane-PNA/DNA is estimated to be 30:1.

**Figure 9:**
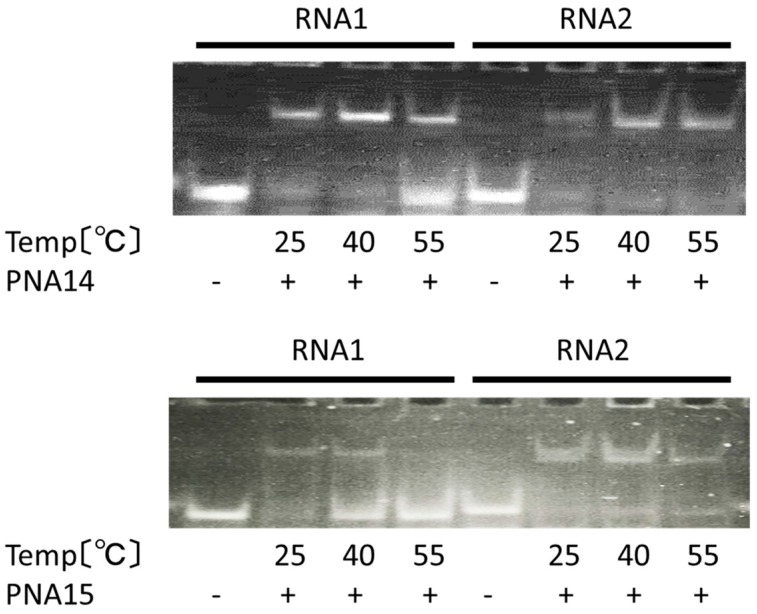
Gel mobility shift assay to assess single nucleotide polymorphism recognition in an RNA oligonucleotide (RNA**1**, RNA**2**) by PNA**13** and PNA**14**. RNA**1** contains the partial sequence from neuraminidase inhibitor-sensitive influenza A/Yokohama/1/H1N1, while RNA**2** contains the partial sequence from the neuraminidase inhibitor-sensitive influenza A/Yokohama/77/H1N1. PNA; 300 nM, RNA; 100 nM, incubation; 10 mM phosphate buffer (pH 6.0); and, 1 mM EDTA at room temperature for 10 min, gel shift assay; 15% acrylamide gel, 20 mA, 60 min.

We performed a gel mobility shift assay to study the effect of tolane**9** on the duplex formation between PNA and RNA. Cy5-labelled synthetic RNA oligonucleotides (RNA**1** and RNA**2** at a final concentration 100 nM) were incubated with three equivalents of the PNAs (PNA**13** and PNA**14** at a final concentration 300 nM) for 10 min in 10 mM sodium phosphate buffer and 1 mM EDTA (pH 6.0) at 25 °C, 40 °C, and 55 °C. PNA**13** bound to both the mismatched RNA**1** and the matched RNA**2** and gave band shifts on the gel, regardless of the incubation temperature, as shown in Figure 9. PNA showed low sequence specificity, as a mismatch base is located near the terminal of the target sequence. However, PNA**13** showed less binding to the mismatched RNA**1**, as the incubation temperature was increased to 55 °C. In contrast, PNA**14** showed reduced binding to the mismatched RNA**1** at 40 °C and the band shift was drastically reduced at 55 °C. Tolane-modified PNA showed improved sequence specificity, as a mismatch base is located near the terminal of the target sequence, which was probably due to selective stacking interactions with only the matched neighboring base pair. These results correspond to the results of their melting temperature analyses that are summarized in Table 7. Regarding the *N*-terminus-modified PNA, a lysine-rich cationic peptide-modified PNA was previously reported to increase the binding constant to a matched DNA by up to 250–fold; however, it also increased the binding constant to mismatched DNA sequences by up to 35–fold relative to that of non-modified PNA [24]. Therefore, tolane-PNA is a better option for discriminating SNPs in a target gene.

**Figure 10:**
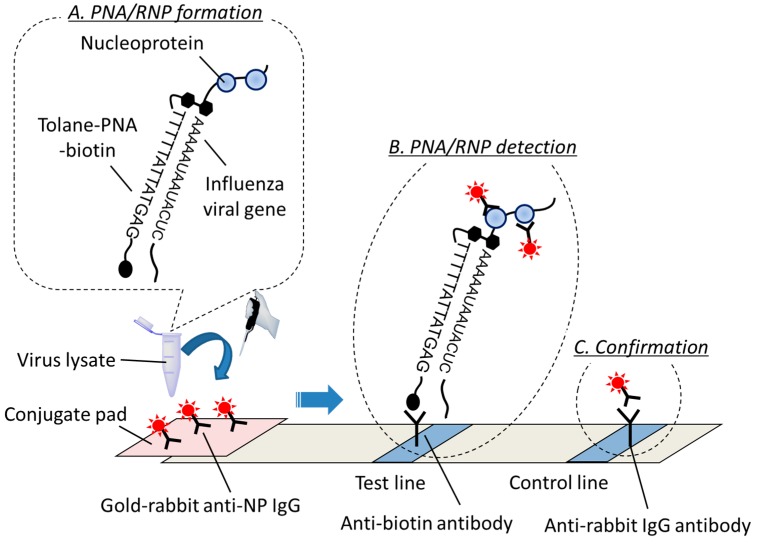

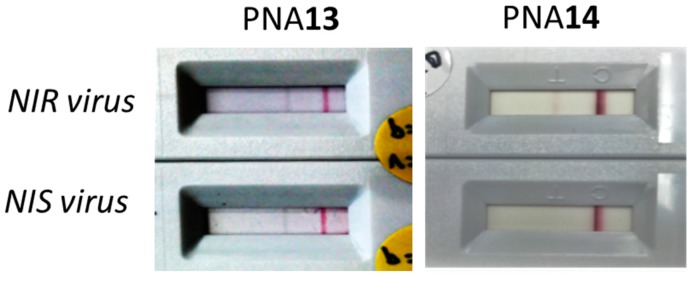
Rapid diagnosis of influenza virus by PNA chromatography. Top) Schematic diagram of the PNA chromatography kit. Top A) PNA/RNP formation, Top B) PNA/RNP detection, Top C) Confirmation. Bottom left) PNA**13** did not discriminate a SNP in the target viral RNA among the two viruses and detected both NIR and NIS viruses on the PNA chromatograph. Bottom right) PNA**14** discriminated a SNP in the target viral RNA among these two viruses and selectively detected NIR virus on the PNA chromatograph. PNA, 5 μL (0.5 μg); influenza viruses, 10 μL (1.0 × 10^6^ pfu/mL); elution buffer, 105 μL (containing 1% Tween, 0.5% BSA). PNA and virus lysate incubation: 55 °C, 5 min. Chromatography incubation: 55 °C, 15 min.

Here, we proposed a new PNA chromatography system to detect a SNP related to the neuraminidase-inhibitor susceptibility of the influenza viral gene. We recently reported a PNA-based ELISA system [25] and a rapid diagnostic test kit while using nucleic acid chromatography for discriminating influenza A viruses [26]. In both methods, PNAs target a conserved sequence of a viral gene of influenza A viruses. These PNA recognizes the viral gene in a sequence-specific manner. As the viral RNA forms ribonucleoprotein complexes with nucleoprotein (NP) [27], their PNAs can form a complex with viral RNA/nucleoprotein, or ribonucleoprotein (RNP) within the virus lysate (Figure 10, top A; PNA/RNP formation). This PNA/RNP complex can be visualized by a gold nanoparticle-conjugated rabbit anti-nucleoprotein IgG antibody (gold-rabbit anti-NP IgG) in the control line, and a red color develops as a result of surface plasmon resonance (Figure 10, top B; PNA/RNP detection). In addition, we prepared a control line that can capture gold-rabbit anti-NP IgG by anti-rabbit IgG antibody to confirm the flow of the sample through the device (Figure 10, top C; Confirmation). We employed this system to discriminate a SNP that is involved in neuraminidase inhibitor resistance and neuraminidase inhibitor-sensitive viral strains.

We decided to perform this nucleic acid chromatography detection at 55 °C to eliminate mismatched binding but to retain matched binding since PNA**14** can slightly bind to RNA**1** that contains a mismatched base at 40 °C (Figure 9). First, we added a neuraminidase inhibitor-resistant virus (NIR virus) and neuraminidase inhibitor-sensitive virus (NIS virus) into an elution buffer (see Materials and Methods section). Subsequently, PNA**13** or PNA**14** was added to the elution mixture and then incubated at 55 °C for 5 min. The PNA-containing elution mixtures were dropped onto a conjugation pad of the PNA chromatography at 55 °C and let react for 15 min. As a result, PNA**13** could not discriminate the SNPs between these two viruses and detected both the NIR and the NIS viruses on the chromatograph (Figure 10, bottom left). By contrast, PNA**14** discriminated the SNPs among these viruses and selectively detected the NIR virus, which possesses a complementary viral RNA sequence, on the chromatograph (Figure 10, bottom right). These results correspond to the results of the gel mobility shift assay that is shown in Figure 9. Further, our chromatography system is advantageous when compared to microarray technology, since it does not require the additional steps of target genome amplification, hybridization, washing, and fluorescence detection during the diagnosis process. Our chromatography method is also advantageous when compared to the immunochromatography approach that has been widely used in bedside applications. In particular, the immunochromatography kit requires two antibodies for the detection of a target antigen in a sandwich fashion and, thus, the sensitivity and specificity rely on the quality of these two antibodies. By contrast, PNA chromatography requires a tolane-PNA that recognizes a conserved gene sequence of the target pathogen and an antibody recognizes the nucleoproteins that are associated with the target gene. The design and synthesis of a certain tolane-PNA for detecting a viral or bacterial gene can be accomplished within one week. The development of an antibody targeting an antigen (i.e., nucleoprotein) is easier than developing two antibodies. One of the drawbacks of tolane-PNA chromatography is its low sensitivity (detection limit: 1.0 × 10^6^ pfu/mL). In addition, some binding was lost to the matched binding since a relatively high incubation temperature is required to eliminate the mismatched binding. However, taking these advantages of tolane-PNA together, we could succeed in developing a novel type of nucleic acid chromatography that can effectively discriminate a SNP in influenza A virus.

## 3. Materials and Methods

### 3.1. Chemicals for Synthesis of Tolane Derivatives and General Analysis

All of the reagents and dry solvents were used without any further purification. Silica gel 60N (spherical, neutral, particle size 40–50 um, Kanto Chemical Co. Inc., Tokyo, Japan) were used for column chromatography, unless otherwise noted. Thin-layer chromatography was performed while using Merck TLC silica gel 60 F254 (Merck KGaA, Darmstadt, Germany). Nuclear magnetic resonance (NMR) spectrum was recorded on a JEOL JML-LA-400 H1 400MHz (JEOL Ltd., Tokyo, Japan). The spectra were internally referenced to the tetramethylsilane signal at 0 ppm, CDCl3 (7.24 ppm), and DMF (2.5 ppm). Mass measurements were recorded on JEOL JMS-T100LC (ESI-TOF-HRMS) (JEOL Ltd., Tokyo, Japan).

### 3.2. Synthesis of Tolane Derivatives

The details were written in the supplemental data. Briefly, we prepare the linker molecule with a carboxylic acid one side and 4-bromo-phenyl group the other side. The carboxylic acid was esterified with methanol and 4-bromo-phenyl group was reacted with ethynylbenzene while using the Sonogashira coupling reaction. After the methoxy ester was hydrolysed to carboxylic acid in lithium hydroxide solution. The free carboxylic acid group was used for amide coupling with the N-terminal amino of PNA.

### 3.3. Chemicals for PNA Synthesis

Fmoc/Bhoc-protected PNA monomers were purchased from Panagene (Daejeon, Korea). Fmoc-Lys(Boc)-OH, poly-ethylene linker, and TGR-resin were purchased from Merck Millipore (Tokyo, Japan). The coupling activators and HATU were purchased from Watanabe Chemicals (Hiroshima, Japan). DNA (salt free) was purchased from Sigma–Genosys (Ishikari, Japan). Other chemicals were purchased from Wako Pure Chemical (Osaka, Japan), Sigma–Aldrich (Tokyo, Japan), and Tokyo Chemical Industry (Tokyo, Japan). The reagents and solvents were used without further purification, unless otherwise noted.

### 3.4. Preparation of Fmoc-Lys-(Boc)-OH Loaded Resin

The Novasyn TGR resin (200 mg, 0.24 mmol/g) was swollen in 5 mL DMF for 30 min prior to the synthesis. 200 μL of base solution (0.3 M 2,6-lutidine, 0.2 M diisopropyl ethylamine, and 0.33 M thiourea solution) and 0.5 M HATU were added to a mixture of Fmoc/Boc-protected lysine (Fmoc-Lys(Boc)-OH, 22.5 mg, 48 μmol), and Boc/Cbz-protected lysine (Boc-Lys(Cbz)-OH, 72.3 mg, 190 μmol) in 200 μL of DMF to activate the carboxyl groups of coupling monomers. For the coupling reaction, the mixture was added to the resin and then incubated at ambient temperature for 60 min. The resin was then removed from the reaction solution and washed with DMF (5 × 5 mL). For the capping of non-reacted amine groups, the resin was treated with 1.5 mL of capping solution (2,6-lutidine: Ac_2_O:pyridine = 6:5:89, *v*/*v*) for 5 min and washed with DMF (10 × 5 mL). For the deprotection of Fmoc-groups, the resin was treated with 1 mL of deblock solution (40% (*v*/*v*) piperidine in DMF) for 5 min and washed with DMF (10 × 5 mL).

### 3.5. PNA Synthesis

Automated linear solid phase synthesis of PNA was performed while using an Intavis ResPep parallel synthesizer that was equipped with micro scale columns (Köln, Germany). The lysine-loaded resin (200 mg, 16 μmol) was swollen in 5 mL DMF for 30 min, and 20 mg of the resin was transferred to each column in the synthesizer. After the removal of DMF, the Fmoc-protecting groups of the lysine-loaded resin were removed from the resin by a 10-min incubation in 100 μL of deblock solution; the resin was subsequently washed with 100 μL of DMF 10 times. The concentration of each monomer (Fmoc-PNA monomers, Fmoc-AEEA-OH, Fmoc-AZO-OH, and Fmoc-Lys(Boc)-OH) was adjusted to 0.3 M in DMF solution. The activator solution contained 0.5 M HATU in DMF. The base solution contained 0.3 M 2,6-lutidine, 0.2 M diisopropyl ethylamine, and 0.33 M thiourea in 5% NMM in pyridine solution (*v*/*v*). The deblock solution contained 40% pyperidine in DMF solution.

For the coupling reaction, 17.5 μL of monomer solution, 17.0 μL of activation solution, and 8.50 μL of base solution were combined in a vessel and incubated for 2 min at ambient temperature. The coupling solution was then transferred to 20 mg of the resin and then incubated for 100 min at ambient temperature. After eluting the coupling solution from the resin by filtration, the resin was washed with 100 μL of DMF 10 times. This coupling procedure was repeated twice for each monomer elongation reaction. The resin was then incubated with 100 μL of capping solution for 10 min to protect the non-elongated amino groups with acetyl groups and subsequently washed with 100 μL of DMF 10 times. The N-terminus Fmoc-group was then removed by incubating the resin with 100 μL of deblock solution for 10 min and subsequently washing with 100 μL of DMF 10 times. The coupling step of the next monomer and capping steps were repeated, as described above, until the desired PNA molecule was synthesized. Before the cleavage of PNA molecules from the resin, the resin was washed with 100 μL of DMF five times, followed by washing with 100 μL of dichloromethane five times. After drying the resin, 1 mL of TFA/m-cresol (9:1, *v*/*v*) was added and incubated for 12 h. The resin was then filtered and the flow-through containing the cleaved PNA was transferred to a new tube. The PNA solution was added to 15 mL of ice-cold diethyl ether, and the precipitate was collected by centrifugation at 4400 rpm for 4 min. The supernatant was transferred to another tube and then discarded. The residue was dried under ambient atmosphere and then dissolved in 100 μL of distilled water.

### 3.6. PNA Purification and Analysis

All of the PNAs were purified by reverse-phase HPLC using a JASCO PU-2086 pump system (Tokyo, Japan) with a JASCO UV-2075 detector and a GL Science Inertsil (150 mm × 4.6 mm, 5 μm) C-18 column for analytical runs or a GL Science Inertsil (20 mm × 250 mm, 3 μm) C-18 column for semi-preparative runs. Eluting solvents (analytical: A (0.1% TFA in water) and B (0.1% TFA in acetonitrile); semi preparative: A (0.1% TFA in water) and B (0.1% TFA in acetonitrile)) were used in a linear gradient at a flow rate of 1 mL/min for analytical and 5 mL/min for semi-preparative HPLC. The gradient for analytical runs was 0→50% B in 30 min, and the gradient for semi-preparative runs was 0% B for 10 min, 0→5% B in 10 min, 5% B for 10 min, 5→10% B in 10 min, 10% B for 10 min, 10→20% B in 120 min, and 20→50% B in 10 min. Detection was performed while using a UV-VIS-detector at 260 nm. PNA molecular weights were analysed using an Ultraflextreme MALDI TOF Mass Spectrometer (Bruker Daltonics, Yokohama, Japan). The optical densities of PNA and DNA were measured at 260 nm with a UV1700 spectrometer (Shimadzu, Kyoto, Japan) using quartz cuvettes (4 × 10 mm). The extinction coefficient of PNA was calculated from the molar extinction coefficient obtained from http://www.panagene.com/. The molar extinction coefficient of tolane derivatives **1**–**8**, **9**, **10**, **11**, and **12** were 15,600, 15,000, 16,500, 9600, and 11,000 (M^−1^cm^−1^), respectively. Measurements of absorption at 260 nm were carried out in a buffer solution (10 mM NaH_2_PO_4_, pH 7.0) at an ambient temperature.

### 3.7. UV-Melting Analysis of PNA/DNA and Tolane-PNA/DNA

PNAs were preheated at 95 °C for 5 min to prevent aggregation, and then gradually cooled to 25°C before being added to the DNA solution. The melting profiles of PNA complexed with DNA or RNA were analysed on a UV1700 spectrophotometer (Shimadzu) while using a microcell (eight cells, 1 mm) at 260 nm. PNAs and DNA were suspended in 20 mM sodium phosphate buffer (pH 7.4) at 4 µM each. The PNAs and RNA were suspended in 10 mM sodium phosphate buffer and 1mM EDTA (pH 6.0) at 4 µM each. The temperature was ramped down from 95 °C to 10 °C at a rate of −1 °C/min.

### 3.8. In silico Conformational Search of Tolane Derivatives in the PNA/DNA Duplex

A PNA/DNA duplex structure that was solved by NMR methodology has been registered in the Protein Data Bank (PDB ID:1PDT). Tolane derivatives were introduced to the N-terminal amino group of PNA via an amide linkage, and an *in silico* conformational search of tolane within the PNA/DNA duplex was performed by Macromodel version 10.5 (Schrödinger, LLC, New York, NY, 2014). The torsion angle search approach, followed by minimization using an OPLS-2005 force field in water was utilized to analyze the stacking conformers.

### 3.9. Analysis of Duplex Conformation of Tolane-PNA/DNA Duplexes by Fluorescence Spectroscopy

Cy5-labelled DNA concentrations were quantified by absorbance at 260 nm while using a molar extinction coefficient provided by manufacturer. Fluorescence Spectrophotometer F-7000 identified excitation wavelength and emission wavelengths at 323 nm and 384 nm. PNA**9** were preheated at 95 °C for 5 min to prevent aggregation, then gradually cooled to 25 °C before being added to DNA solutions. PNA**9**, DNA**1**, and DNA**2** concentrations were quantified by absorbance at 260 nm while using a molar extinction coefficient that was provided by the manufacturer. Fluorescence spectrum of PNA**9** alone, PNA**9**/DNA**1**, and PNA**9**/DNA**2** were measured in 20 mM sodium phosphate (pH 7.4) at 25 °C.

### 3.10. Gel Mobility Shift Analysis of PNA/RNA Complexes

PNAs were preheated at 95 °C for 5 min to prevent aggregation, and then gradually cooled to 25 °C before being added to the RNA solution. The RNA concentrations were quantified by absorbance at 260 nm while using a molar extinction coefficient provided by the manufacturer. PNA/RNA hybridization assays were conducted while using 100 nM Cy5-labeled single strand RNA with 300 nM PNA, or tolane-PNA in 10 mM sodium phosphate and 1 mM EDTA at pH 6.0 for 10 min at 25 °C. The reaction mixture for each condition was mixed with 0.2 volumes of a solution containing 30% glycerol, 0.025% bromophenol blue, and 0.025% xylencyanol (Sigma-Aldrich Japan, Tokyo, Japan), and then subjected to electrophoresis at 20 mA for 60 min on a 15% non-denaturing polyacrylamide gel using 1x TBE as a running buffer (89 mM Tris base, 89 mM borate, 2 mM EDTA, pH 8.1) at 4 °C in the dark. The gel images were created by the use of a CCD digital image stock system, FAS-III (Toyobo, Osaka, Japan).

### 3.11. Preparation of Influenza A/Yokohama/1/2008/H1N1 and A/Yokohama/77/2008/H1N1

Influenza A/Yokohama/1/2008/H1N virus and A/Yokohama/77/2008/H1N virus were propagated in chicken embryonated eggs and then purified by sucrose gradient ultracentrifugation. For the PNA binding assay, the viral titers were assessed while using a plaque formation assay and adjusted to 1.0 × 10^6^ pfu/mL with a phosphate buffered saline (pH 7.4).

### 3.12. Discrimination of a SNP in the Neuraminidase Gene of Influenza A Virus by PNA and Tolane-PNA Using Nucleic Acid Chromatography

The samples containing influenza virus (10 μL of 1.0 × 10^6^ pfu/mL) were added to 105 μL of phosphate buffer (pH 7.4) containing detergents at 55 °C and dropped to a conjugate pad of the kit. We first formed the complex between tolane-PNA-biotin and influenza viral RNA/nucleoprotein in the sample lysate, which was dropped onto the conjugation pad, and then flowed by chromatography on the membrane towards the test and control lines. PNA-Lys-O-O-Lys(biotin) and tolane-PNA-Lys-O-O-Lys(biotin) was preheated at 95 °C for 5 min to prevent aggregation, and then gradually cooled to 55 °C before being added to the influenza virus detergent. After incubation of 0.5 μg of PNA-Lys-O-O-Lys(biotin) and tolane-PNA-Lys-O-O-Lys(biotin) solution with 115 μL of the viral lysate at 55 °C for 5 min, the mixture was dropped onto a conjugate pad on a lateral flow strip that contains a gold-rabbit anti-nucleoprotein at 55 °C in a CO_2_ gas incubator SCA series (ASTEC, Fukuoka, Japan). PNAs can form a complex with the influenza viral RNA and the nucleoprotein (RNP) within the virus lysate. This PNA/RNP complex can be visualized by gold-nanoparticle that was conjugated rabbit anti-nucleoprotein IgG antibody (gold-rabbit anti-NP) in the control line, and a red color develops as a result of surface plasmon resonance. In addition, we prepared a control line that can capture the excess gold-rabbit anti-NP by anti-rabbit IgG to confirm the flow of sample through the device. We employed this system to discriminate a SNP in neuraminidase inhibitor-resistant and neuraminidase inhibitor-sensitive viral strains.

## 4. Conclusions

We designed and synthesized various types of tolane derivatives that possess different types of linkers, such as alkyl linkers, ether linkers, and amide linkers. These were incorporated at the N-terminus of the PNA to increase its sequence specificity against ssDNA. As a result, we found that the best linker length among the different alkyl acids tested was pentanoic acid. The substitution of pentanoic acid with ethoxypropanoic acid further increased the binding affinity of the tolane-PNA due to its flexible structure. Extension of the pi-conjugated system by changing from a phenyl to naphthyl group also increased the binding affinity to neighboring base pairs and increased duplex stability in a sequence specific manner. We were able to discriminate a single base mismatch in the terminal region of target DNA and RNA molecules without increasing the binding to mismatched genes to activate the carboxyl groups of coupling monomers. A novel type of nucleic acid chromatography that employed our tolane-modified PNA allowed for us to detect a SNP related to drug-resistance found in an influenza virus without using PCR-based genome amplification reaction. Therefore, tolane-modified PNA chromatography has the potential to directly enable the simple diagnosis of drug-resistant viruses from viral samples at the bedside without requiring PCR, gel electrophoresis, mass spectroscopy, or fluorescence detection.

## Figures and Tables

**Table 1:** PNA and DNA sequences used for studying the effect of tolane molecules on UV-melting temperature analysis.

Name	PNA (N-C)/DNA or RNA (5′-3′)	Mass	
		Calculated	Found
**PNA0**	TTCCCTCCTCTA-Lys	3258.38	3261.74
**PNA1**	Tolane**1**-TTCCCTCCTCTA-Lys	3490.67	3495.84
**PNA2**	Tolane**2**-TTCCCTCCTCTA-Lys	3476.65	3482.33
**PNA3**	Tolane**3**-TTCCCTCCTCTA-Lys	3504.70	3508.71
**PNA4**	Tolane**4**-TTCCCTCCTCTA-Lys	3518.73	3519.78
**PNA5**	Tolane**5**-TTCCCTCCTCTA-Lys	3532.76	3536.00
**PNA6**	Tolane**6**-TTCCCTCCTCTA-Lys	3520.70	3520.79
**PNA7**	Tolane**7**-TTCCCTCCTCTA-Lys	3520.70	3521.16
**PNA8**	Tolane**8**-TTCCCTCCTCTA-Lys	3533.72	3534.27
**PNA9**	Tolane**9**-TTCCCTCCTCTA-Lys	3570.76	3570.68
**PNA10**	Tolane**10**-TTCCCTCCTCTA-Lys	3644.85	3642.05
**PNA11**	Tolane**11**-TTCCCTCCTCTA-Lys	3550.73	3552.13
**PNA12**	Tolane**12**-TTCCCTCCTCTA-Lys	3545.71	3546.78
**DNA1**	ATGTCCTAGAGGAGGGAATAA	-	-
**DNA2**	ATGTCCTAGAGGAGGGCATAA	-	-

Lys: lysine, Underlined: mismatch base. Calculated: expected molecular weight, Found: molecular weight identified by MALDI-TOFMS.

**Table 2:** Thermal stability of PNA/DNA and tolane-PNA/DNA duplexes.

PNA	Tm (°C)		
	Match ^1^	Mismatch ^2^	∆Tm ^3^
0	56.5 ± 0.9	48.6 ± 0.9	7.9
1	60.8 ± 0.6	48.8 ± 0.7	12.0
2	59.3 ± 0.9	48.5 ± 0.9	10.8
3	60.1 ± 0.1	48.1 ± 0.7	12.0
4	61.7 ± 0.9	48.7 ± 0.8	13.0
5	59.6 ± 0.2	48.4 ± 0.3	11.2
6	63.0 ± 0.6	48.5 ± 0.9	14.5
7	62.2 ± 0.3	48.8 ± 0.6	13.4
8	60.9 ± 0.3	48.7 ± 0.4	12.2
9	64.8 ± 0.6	48.3 ± 0.9	16.5
10	60.7 ± 1.2 ^4^	48.6 ± 0.3 ^4^	12.1 ^4^
11	60.9 ± 0.5	48.9 ± 0.7	12.0
12	63.3 ± 0.7	49.2 ± 0.2	14.1

^1^ Mean Tm ± SD (n = 3), Tm between each PNA and DNA**1**, ^2^ Mean Tm ± SD (n = 3), Tm between each PNA and DNA**2**. ^3^ ΔTm = Tm(matched) − Tm(mismatched). ^4^ 20% methanol was added to dissolve the pyrene-PNA in 20 mM phosphate buffer (pH 7.4).

**Table 3:** Thermodynamic parameters of the PNA/DNA duplex.

	∆G°(298K) (kcal/mol)	∆H° (kcal/mol)	∆S°(298K) (cal/mol·K)	Tm °C
PNA**0**/DNA**1**	−13.7	−61.8	−159.5	56.6 ± 0.9
PNA**9**/DNA**1**	−16.9	−79.1	−208.8	64.9 ± 0.6
PNA**0**/DNA**2**	−10.7	−44.6	−113.7	49.4 ± 0.9
PNA**9**/DNA**2**	−11.3	−47.6	−121.5	50.2 ± 0.9

**Table 4:** PNA and DNA sequences used to study single base mismatch discrimination. The underlined sequences in DNA**3**, **4**, and **5** were the mismatched bases to both PNA**0** and PNA**9**.

	PNA (N-C)/DNA (5′-3′)
PNA**0**	TTCCCTCCTCTA-Lys
PNA**9**	Tolane9-TTCCCTCCTCTA-Lys
DNA**1**	ATGTCCTAGAGGAGGGAATAA
DNA**3-T**	ATGTCCTAGAGGAGGGATTAA
DNA**3-G**	ATGTCCTAGAGGAGGGAGTAA
DNA**3-C**	ATGTCCTAGAGGAGGGACTAA
DNA**4-T**	ATGTCCTAGAGGAGGGTATAA
DNA**4-G**	ATGTCCTAGAGGAGGGGATAA
DNA**4-C**	ATGTCCTAGAGGAGGGCATAA
DNA**5-T**	ATGTCCTAGAGGAGGTAATAA
DNA**5-A**	ATGTCCTAGAGGAGGAAATAA
DNA**5-C**	ATGTCCTAGAGGAGGCAATAA

**Table 5:** UV-melting temperature analysis of PNA**0** and PNA**9** with DNA containing a single base mismatch at the first (DNA**3**), second (DNA**4**), and third (DNA**5**) base from the N-terminus of the PNA.

PNA	Tm (°C)									
	Matched ^1^ DNA1	Mismatched ^1^ DNA3			Mismatched ^1^ DNA4			Mismatched ^1^ DNA5		
	T/A	T/T	T/G	T/C	T/T	T/G	T/C	C/T	C/A	C/C
PNA0	56.6 (±0.9)	57.0 (±0.4)	57.7 (±0.3)	53.0 (±0.9)	56.7 (±1.9)	56.6 (±1.2)	49.6 (±0.9)	54.0 (±0.8)	53.7 (±0.7)	52.5 (±1.1)
PNA9	64.9 (±0.6)	59.5 (±0.1)	60.1 (±0.3)	53.3 (±0.2)	58.5 (±0.8)	59.2 (±0.9)	50.2 (±0.9)	55.4 (±0.4)	55.2 (±0.5)	52.8 (±0.5)

^1^ Mean Tm ± SD (n = 3), PNA, DNA; 4 μM each in 20 mM phosphate buffer (pH 7.4).

**Table 6:** PNA**13** and PNA**14** used to detect a neuraminidase inhibitor-resistant gene in influenza virus A/Yokohama/77/H1N1 derived from a single nucleotide polymorphism (SNP) within the sequence. RNA**1** contains the neuraminidase inhibitor-sensitive gene in influenza virus A/Yokohama/1/2008/H1N1, while RNA**2** contains the neuraminidase inhibitor-resistant gene in influenza virus A/Yokohama/77/2008/H1N1. DNA**6** and DNA**7** contain the same sequence as RNA**1** and RNA**2**, except that uracil bases were substituted with thymine bases.

Name	PNA (N-C)/RNA (5′-3′) or DNA	Mass	
		Calculated.	Found
**PNA13**	TTTTATTATGAG-Lys-*O*-*O*-Lys-biotin	4062.35	4063.27
**PNA14**	Tolane9-TTTTATTATGAG-Lys-*O*-*O*-Lys-biotin	4374.45	4374.45
**DNA6**	GCATTCCTCATAATA**G**AAATT	-	-
**DNA7**	GCATTCCTCATAATAAAAATT	-	-
**RNA1**	Cy5-GCAUUCCUCAUAAUA**G**AAAUU	-	-
**RNA2**	Cy5-GCAUUCCUCAUAAUAAAAAUU	--	-

O: aminoethylethoxyacetate.

**Table 7:** UV-melting temperature analysis of PNA/DNA and PNA/RNA containing the neuraminidase inhibitor-resistant and neuraminidase-sensitive gene sequences.

	Tm^1^ (°C) With Sensitive Sequence	Tm^2^ (°C) With Resistant Sequence	ΔTm (Tm^2^ − Tm^1^) (°C)
PNA**13**	48.0 (DNA6)	53.5 (DNA7)	5.5
PNA**14**	47.6 (DNA6)	59.3 (DNA7)	11.7
PNA**13**	40.2 (RNA1)	51.7 (RNA2)	11.5
PNA**14**	31.5 (RNA1)	55.3 (RNA2)	23.8

PNA/RNA; 4 μM each in 10 mM phosphate buffer and 1 mM EDTA (pH 6.0).

## Supplementary Materials

The following are available online, Figure S1: Conformational search of tolane**4**-PNA/DNA duplex.
