# Supplementary material for: SNP Discrimination by Tolane-Modified Peptide Nucleic Acids: Application for the Detection of Drug Resistance in Pathogens
Source: Molecules. 2020 Feb 11;25(4):769. doi: 10.3390/molecules25040769 (PMC7070780; doi:10.3390/molecules25040769)
Supplement: Supplementary file 1 [file molecules-25-00769-s001.pdf]

SNP Discrimination by Tolane-Modified Peptide Nucleic Acids: Application for the Detection of Drug Resistance in Pathogens

Kenji Takagi[1], Tenko Hayashi[1], Sinjiro Sawada[1], Miku Okazaki[1], Sakiko Hori[1], Katsuya Ogata[1], Nobuo Kato[1], Yasuhito Ebara[2*], Kunihiro Kaihatsu[1,2*]

*Corresponding author.

Supporting Information

Table of Contents: S2

Supplementary Figures

Figure S1 S3

Supplementary data

Synthesis of tolane derivatives S4-17
PNA data analysis S18-24
Abbreviations S25
Reference S26

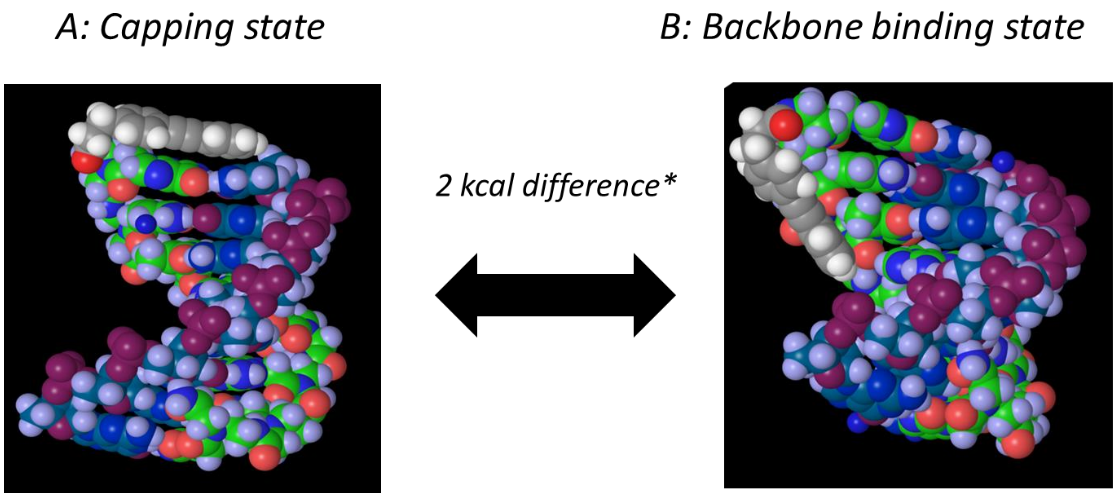

Figure S1. Conformational search of tolane**4**-PNA/DNA duplex. The PNA/DNA duplex structures were obtained as 1PDT from the PDB data bank. Gray; tolane**4**, Green; PNA backbone, Purple; DNA backbone, Orange; PNA**8**. Left: Tolane interacted to the adjacent PNA/DNA base pair and formed "capped state" with adjacent base pairs. Right: Tolane interacted to the PNA backbone and formed "Backbone binding state". and the conformational search was performed using the force field OPLS2005 model and MacroModel. A capped state is 2 kcal stable than backbone binding state, which indicates the expected molar ratio between capped state and backbone binding state is 30:1.

Methyl 3-(4-bromophenyl)propanoate (1a)

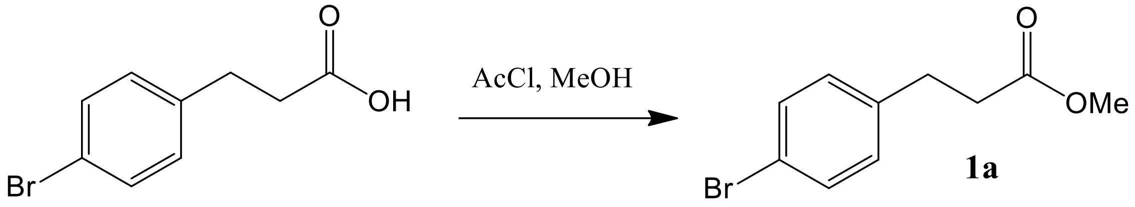

Acetyl chloride (3.8 mL, 41.9 mmol) was dropped into 40 ml of methanol at 0 °C and stirred for ten minutes under Ar. 3-(4-Bromophenyl)propanoic acid (4.0 g, 52.4 mmol) was added to the solution, and stirred at room temperature for two hours. The solution was neutralized with potassium carbonate and evaporated under reduced pressure. The residue was dissolved in ethyl acetate, washed with water and brine, and the organic layer was dried over magnesium sulfate. The solvent was evaporated to yield the product (4.35g, quant). 1H-NMR (400 MHz, CDCl3, δ): 7.42-7.39 (m, 2H), 7.09-7.06 (m, 2 H), 3.66 (s, 3H), 2.90 (t, 2H), 2.61 (t, 2H)

Methyl 3-(4-((trimethylsilyl)ethynyl)phenyl)propanoate (1b)

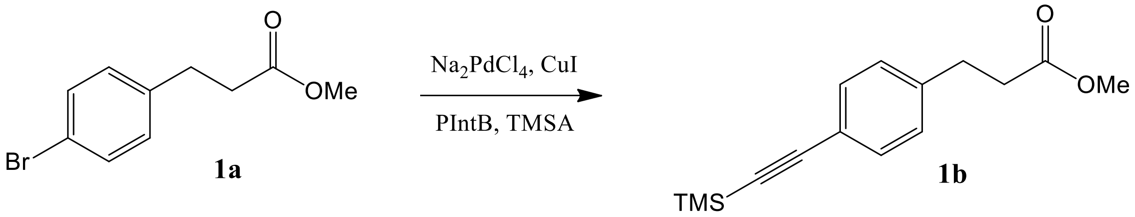

Methyl 3-(4-bromophenyl)propanoate (**1a**) (1.0 g, 4.11 mmol) was dissolved in 7mL of N,N'-dimethylformamide (DMF). Copper (I) iodide (156 mg, 0.82 mmol), triethylamine (TEA 1.2mL, 8.23mmol), tetrakis (triphenylphosphine) palladium (0) （$Pd(PPh_3)_4$ 475 mg, 0.41 mmol）, trimethylsilylacetylene (TMSA 0.74 mL, 5.35 mmol) were added and the reaction mixture was stirred at 80 °C under Ar until all the starting material was consumed, as was monitored by TLC. The reaction mixture was filtered through a pad of celite and organic solvent (ethyl acetate: n-hexane = 10:1) was added. The organic phase was washed two times with saturated ammonium chloride, brine and then dried over magnesium sulfate. The solvent was evaporated to yield the product 740 mg and it was used to next without further purification.

Methyl 3-(4-ethynylphenyl)propanoate (1c)

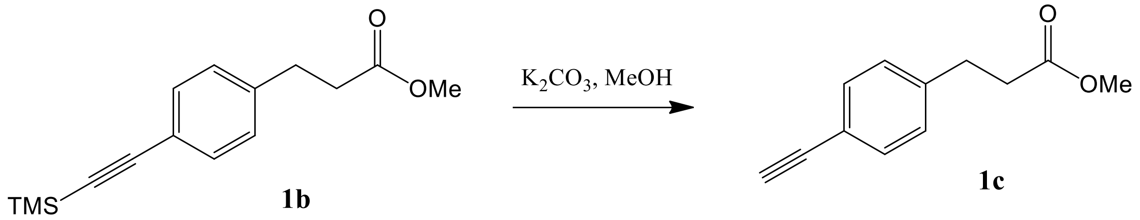

Crude methyl 3-(4-((trimethylsilyl)ethynyl)phenyl)propanoate (**1b**) (740mg) and potassium carbonate 780 mg were added to 10 ml of methanol and stirred at room temperature until all the starting material was consumed, as was monitored by TLC. After water was added to the solution, the aqueous phase was extracted three times with ethyl acetate. The combined organic layers were dried with magnesium sulfate and evaporated under reduced pressure. The residue was purified by column chromatography (hexane : ethyl acetate=5:1(v/v)) to yield the product (243 mg, 31% over 2 steps); 1H-NMR (400 MHz, CDCl3, δ): 7.40 (d, 2H), 7.14 (d, 2 H), 3.64 (s, 3H), 3.06 (s, 1H) 2.90 (t, 2H), 2.61 (t, 2H)

Methyl 3-(4-(phenylethynyl)phenyl)propanoate (1d)

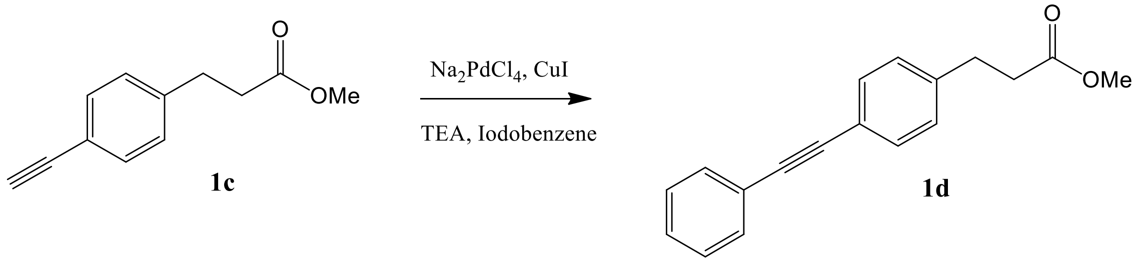

Methyl 3-(4-ethynylphenyl) propanoate (**1c**) (243 mg, 1.29 mmol) was dissolved in 7mL of N,N'- dimethylformamide (DMF). Copper (I) iodide (156 mg, 0.82 mmol), triethylamine (TEA 1.2mL, 8.23mmol), tetrakis (triphenylphosphine) palladium (0) ($Pd(PPh_3)_4$ 475mg, 0.41mmol), trimethylsilylacetylene (TMSA 0.74 mL, 5.35 mmol), and iodobenzene (329mg, 1.61mmol) were added and the reaction mixture was stirred at 80 °C under Ar until all the starting material was consumed, as was monitored by TLC. The reaction mixture was filtered through a pad of celite and organic solvent (ethyl acetate: n-hexane = 10:1) was added. The organic phase was washed two times with saturated ammonium chloride, brine and then dried over magnesium sulfate. The solvent was evaporated under reduced pressure to yield the product (308 mg, 90%); 1H-NMR (400 MHz, CDCl3, δ): 7.51-7.49 (m, 2H), 7.43 (d, 2 H), 7.30-7.26 (m, 3H), 7.12 (d, 2H), 3.60 (s, 3H), 2.89 (t, 2H), 2.56 (t, 2H).

3-(4-(Phenylethynyl)phenyl)propanoic acid (1)

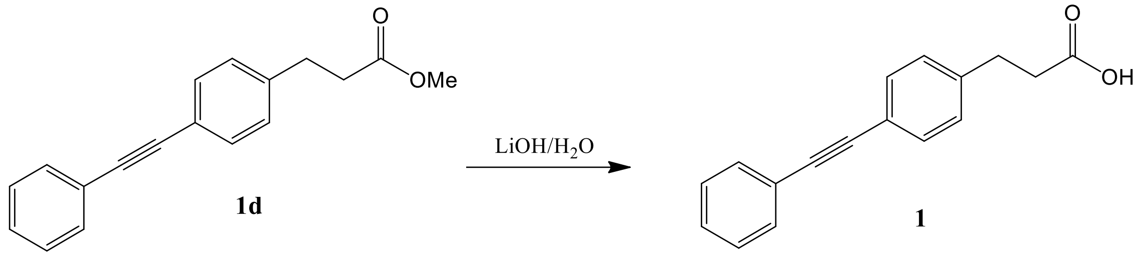

Methyl 3-(4-(phenylethynyl)phenyl)propanoate **(1d)** (308mg, 1.17mmol) was dissolved in 5 mL of THF, and lithium hydroxide monohydrate (98 mg, 2.33 mmol) dissolved in 3 ml of H_2O was added to the solution and stirred at room temperature until all the starting material was consumed, as was monitored by TLC. Water was added to the solution and the pH was adjusted to pH < 1 with 3% HCl solution. The solution was extracted three times with ethyl acetate, washed with brine, and then dried over magnesium sulfate. The solvent was evaporated under reduced pressure to yield the product **2** (248mg, 85%); ^{1}H-NMR (400 MHz, DMSO-d$_6$, δ): 7.53-7.50 (m, 2H), 7.55 (d, 2H), 7.35-7.30 (m, 3H), 7.18 (d, 2H), 2.95 (t, 2H), 2.68 (t, 2H); ESI HRMS calcd for $C_{17}H_{14}O_2$ $[M+Na]^+$: 273.0892, found 273.0889.

Methyl 2-(4-bromophenyl)acetate (2a) [1]

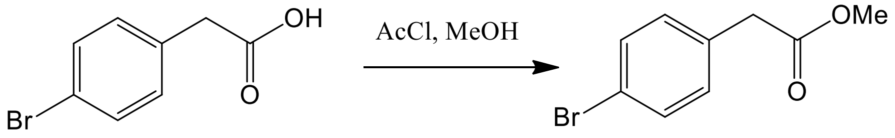

2a

Acetyl chloride (3.0 mL, 41.9 mmol) was dropped into 40 ml of methanol at 0 °C and stirred for ten minutes under Ar. 3-(4-Bromophenyl)propanoic acid (3.0 g, 14.0 mmol) was added to the solution, and stirred at room temperature for two hours. The solution was neutralized with potassium carbonate and evaporated under reduced pressure. The residue was dissolved in ethyl acetate, washed with water and brine, and the organic layer was dried over magnesium sulfate. The solvent was evaporated to yield the product (3.19 g, quant) and used to next step without purification; 1H-NMR (400 MHz, CDCl3, δ): 7.45 (d, 2H), 7.15 (d, 2H), 3.69 (s, 3H), 3.58 (s, 2H)

Methyl 2-(4-(phenylethynyl)phenyl)acetate (2b) [1]

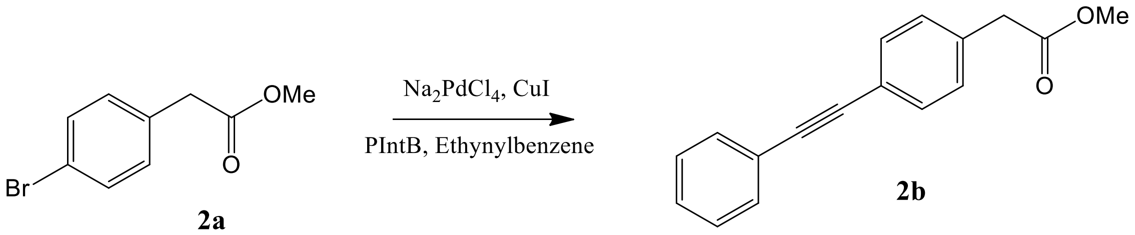

Methyl 2-(4-bromophenyl)acetate (**2a**) (2.0 g, 8.73 mmol) was dissolved in 14 mL of N, N, N', N'- tetramethylethylendiamine (TMEDA) and 1.5 ml of H_2O. Disodium tetrachloropalladate (Na_2PdCl_4, 23mg, 0.08 mmol), Copper (I) iodide (30 mg, 0.16 mmol), 2-(di-*tert*-butylphosphino)-N-phenylindole (PIntB, 62 mg, 0.16 mmol), ethynylbenzene (0.87 mL, 7.94 mmol) was added and the reaction was stirred at 80 °C under Ar until all the starting material was consumed as judged by TLC. Water was added to the solution then extracted three times with ethyl acetate. The organic phase was washed two times with saturated ammonium chloride, brine and then dried over magnesium sulfate. The solvent was evaporated and the residue was purified by column chromatography (hexane : ethyl acetate=4:1(v/v)) to yield the product (1.40 g, 64%).; ^{1}H NMR (400 MHz, $CDCl_3$, δ): 7.54-7.48 (m, 4H), 7.37-7.32 (m, 3H), 7.65 (d, 2H), 3.70 (s, 3H), 3.64 (s, 2H).

2-(4-(Phenylethynyl)phenyl)acetic acid (2) [1]

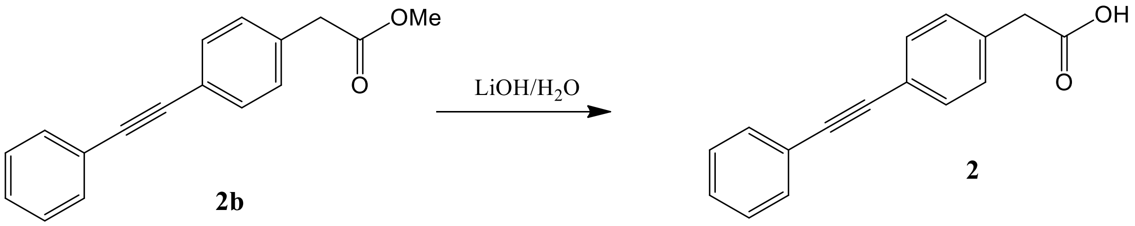

2-(4-(Phenylethynyl)phenyl)acetate (**2b**) (1.40 g, 5.58 mmol) was dissolved in 25ml of THF, and lithium hydroxide monohydrate (470 mg, 11.2 mmol) dissolved in 10 ml of H2O was added to the solution and stirred at room temperature until all the starting material was consumed, as was monitored by TLC. Water was added to the solution and the pH was adjusted to pH < 1 with 3% HCl solution. The solution was extracted three times with ethyl acetate, washed with brine, and then dried over magnesium sulfate. The solvent was evaporated under reduced pressure to yield the product 1 (1.31g, quant); 1H-NMR (400 MHz, DMSO-d6, δ): 7.57-7.55 (m, 2H), 7.51 (d, 2H), 7.45-7.42 (m, 3H), 7.42 (d, 2H), 3.63 (s, 2H); ESI HRMS calcd for $C_{16}H_{12}O_2$ $[M+Na]^+$: 259.0735, found 259.0726.

Methyl 4-(4-bromophenyl)butanoate (3a) [1]

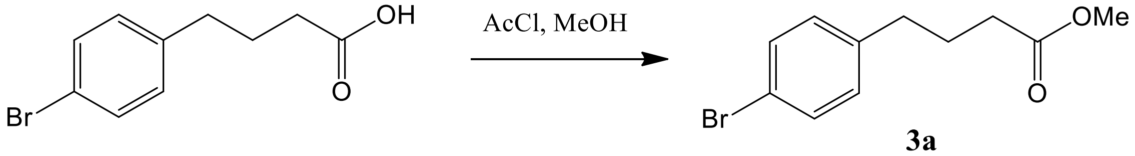

Acetyl chloride (1.8 mL, 24.7 mmol) was dropped into 30 ml of methanol at 0 °C and stirred for ten minutes under Ar. 4-(4-bromophenyl)butanoic acid(2.0g, 8.23mmol) was added to the solution, and stirred at room temperature for two hours. The solution was neutralized with potassium carbonate, and evaporated under reduced pressure. The residue was dissolved in ethyl acetate, washed with water and brine, and the organic layer was dried over magnesium sulfate. The solvent was evaporated to yield the product (2.12 g, quant) and used to next step without further purification; 1H-NMR (400 MHz, CDCl3, δ): 7.41 (d, 2H), 7.05 (d, 2H), 3.67 (s, 3H), 2.60 (t, 2H), 2.32 (t, 2H), 1.93 (q, 2H).

Methyl 4-(4-(phenylethynyl)phenyl)butanoate (3b) [1]

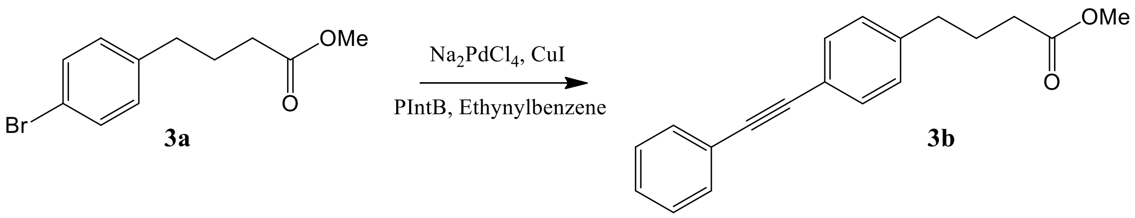

Methyl 4-(4-bromophenyl)butanoate (**3a**) (1.0 g, 3.89 mmol) was dissolved in 6 ml of N, N, N', N'- tetramethylethylendiamine (TMEDA) and 1 ml of H2O. Disodium tetrachloropalladate (Na_2PdCl_4, 10 mg, 0.04 mmol), Copper (I) iodide (14 mg, 0.07 mmol), 2-(di-tert-butylphosphino)-N-phenylindole (PIntB, 28 mg, 0.07 mmol), ethynylbenzene (0.4 mL, 3.53 mmol) was added and the reaction was stirred at 80 °C under Ar until all starting material was consumed, as was monitored by TLC. Water was added to the solution then extracted three times with ethyl acetate. The organic phase was washed two times with saturated ammonium chloride, brine and then dried over magnesium sulfate. The combined organic layers was evaporated and the residue was purified by column chromatography (hexane : ethyl acetate=4:1(v/v)) to yield the product (745mg, 71%); 1H-NMR (400 MHz, CDCl3, δ): 7.51-7.49 (m, 2H), 7.43 (d, 2H), 7.27-7.24 (m, 3H), 7.08 (d, 2H), 3.58 (s, 3H), 2.56 (t, 2H), 2.24 (t, 2H), 1.87 (q, 2H).

4-(4-(phenylethynyl)phenyl)butanoic acid (3) [1]

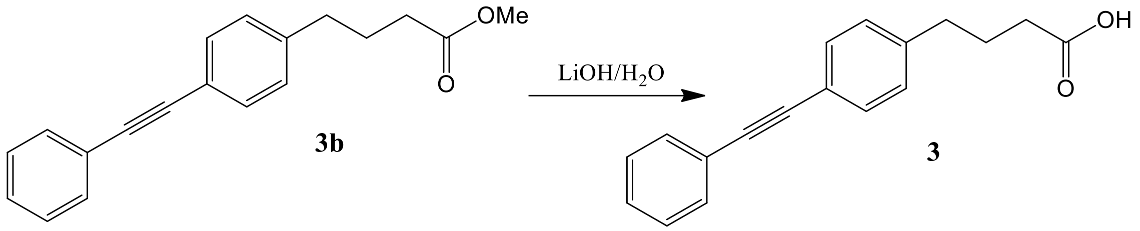

Methyl 4-(4-(phenylethynyl)phenyl)butanoate (**3b**) (745 mg, 2.68 mmol) was dissolved in 13 mL of THF, and lithium hydroxide monohydrate (225 mg, 5.35 mmol) dissolved in 5 ml of H_2O was added to the solution and stirred at room temperature until all the starting material was consumed, as was monitored by TLC. Water was added to the solution and the pH was adjusted to pH < 1 with 3% HCl solution. The solution was extracted three times with ethyl acetate, washed with brine, and then dried over magnesium sulfate. The solvent was evaporated under reduced pressure to yield the product **3** (700 mg, 99%); ^{1}H-NMR (400 MHz, DMSO-d_6, δ): 7.56-7.54 (m, 2H), 7.48 (d, 2H), 7.44-7.41 (m, 3H), 7.26 (d, 2H), 2.63 (t, 2H), 2.23 (t, 2H), 1.81 (q, 2H) ; ESI HRMS calcd for $C_{18}H_{16}O_2$ $[M+Na]^+$: 287.1048, found 287.1040.

5-(4-Iodophenyl)pentanoic acid (4a)

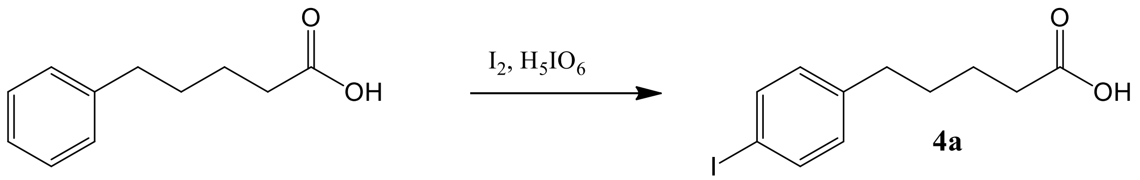

5-Phenylpentanoic acid (1.0 g, 5.61 mmol), H_5IO_6 (260 mg, 1.12 mmol), I_2 (280 mg, 2.24 mmol), 2 ml of 10 M H_2SO_4, 6ml of glacial acetic acid, 1.2 ml of H_2O were stirred at 75 °C under Ar for 18 hours. After the mixture was cooled to room temperature, water was added to the solution. The aqueous phase was washed three times with chloroform and the combined organic layers were washed two times with sodium thiosulfate, brine and dried over magnesium sulfate. The solvent was evaporated under reduced pressure and a small amount of chloroform was added. The product was recrystallized from hexane (405mg, 24%); ^{1}H-NMR (400 MHz, $CDCl_3$, δ): 7.59 (d, 2H), 6.93 (d, 2H), 2.59 (t, 2H), 2.38 (t, 2H), 1.65(q, 4H).

Methyl 5-(4-iodophenyl)pentanoate (4b)

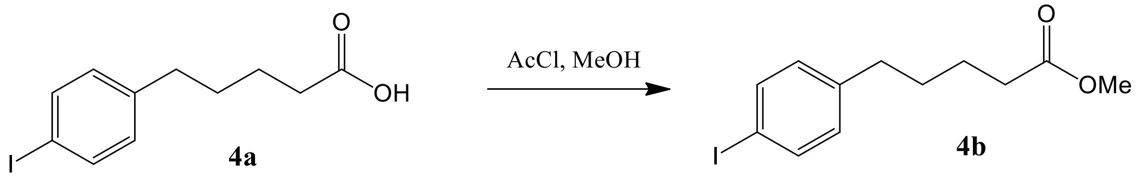

Acetyl chloride (0.3 mL, 4.00 mmol) was dropped into 5 ml of methanol at 0 °C and stirred for

ten minutes under Ar. 5-(4-iodophenyl)pentanoic acid (**4a**) (405 mg, 1.33 mmol) was added to the solution, and stirred at room temperature for two hours. The solution was neutralized with potassium carbonate and evaporated under reduced pressure. The residue was dissolved in ethyl acetate, washed with water and brine, and the organic layer was dried over magnesium sulfate. The solvent was evaporated under reduced pressure to yield the product (445 mg, quant) and used to next step without further purification; ^{1}H-NMR (400 MHz, $CDCl_3$, δ): 7.55 (d,2H), 6.89 (d, 2H), 3.63 (s, 3H), 2.54 (t, 2H), 2.30 (t, 2H), 1.62 (m, 4H)

Methyl 5-(4-(phenylethynyl)phenyl)pentanoate (4c)

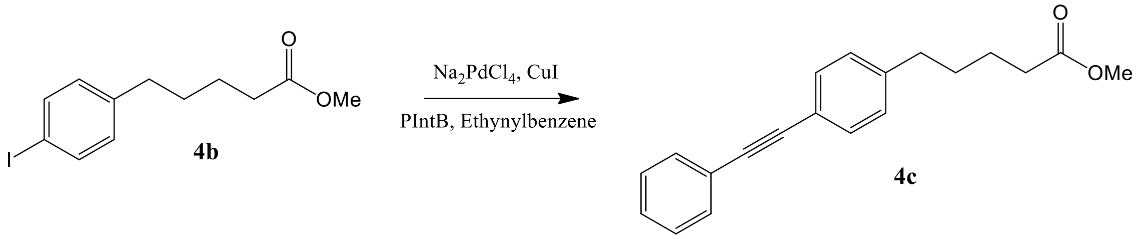

Methyl 5-(4-iodophenyl)petanoate (**4b**) (445 mg, 1.40 mmol) was dissolved in 2.5 ml of N, N, N', N'- tetramethylethylendiamine (TMEDA) and 0.3 ml of H_2O. Disodium tetrachloropalladate (Na_2PdCl_4, 3.7 mg, 0.01 mmol), Copper (I) iodide (5.0 mg, 0.2 mmol), 2-(di-*tert*-butylphosphino)-N-phenylindole (PIntB, 10 mg, 0.02 mmol), ethynylbenzene (0.14 mL, 1.30 mmol) was added and the reaction was stirred at 80 °C under Ar until all starting material was consumed, as was monitored by TLC. Water was added to the solution then extracted three times with ethyl acetate. The combined organic layers were washed two times with saturated ammonium chloride, brine and then dried over magnesium sulfate. The solvent was evaporated under reduced pressure and the residue was purified by column chromatography (hexane : ethyl acetate=4:1(v/v)) to yield the product (395mg, 97%); ^{1}H-NMR (400 MHz, $CDCl_3$, δ): 7.51-7.49 (m, 2H), 7.43 (d、2H), 7.31-7.27 (m, 3H), 7.10 (d, 2H), 3.61 (s, 3H), 2.57 (t, 2H), 2.28 (t, 2H), 1.61 (q, 4H)

5-(4-(Phenylethynyl)phenyl)pentanoic acid (4)

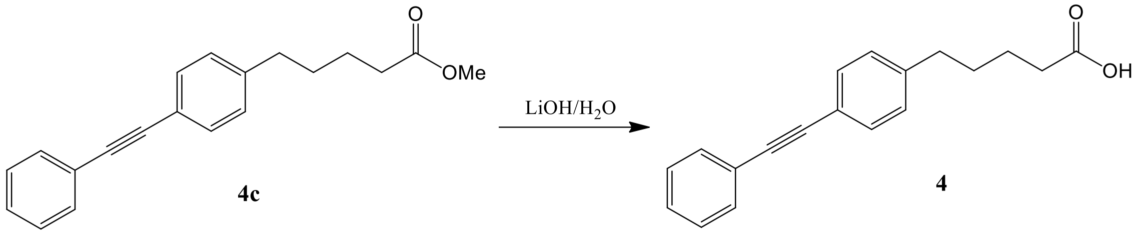

Methyl 5-(4-(phenylethynyl)phenyl)pentanoate (**4c**) (395 mg, 1.35 mmol) was dissolved in 7 mL of THF, and lithium hydroxide monohydrate (113 mg, 2.70 mmol) dissolved in 3 ml of H_2O was added to the solution and stirred at room temperature until all the starting material was consumed, as

was monitored by TLC. Water was added to the solution and the pH was adjusted to pH < 1 with 3% HCl solution. The combined organic layers were extracted three times with ethyl acetate, washed with brine, and then dried over magnesium sulfate. The solvent was evaporated under reduced pressure to yield the product **4** (306mg, 81%); ^{1}H-NMR (400 MHz, $CDCl_3$, δ) : 7.53-7.51 (m, 2H), 7.45 (d、2H), 7.35-7.32 (m, 3H), 7.15 (d, 2H), 2.64 (t, 2H), 2.38 (t, 2H), 1.69 (q, 4H); ESI HRMS calcd for $C_{19}H_{18}O_2$ $[M+Na]^+$: 301.1120, found 301.1198.

6-(4-(Phenylethynyl)phenyl)hexanoic acid (5)

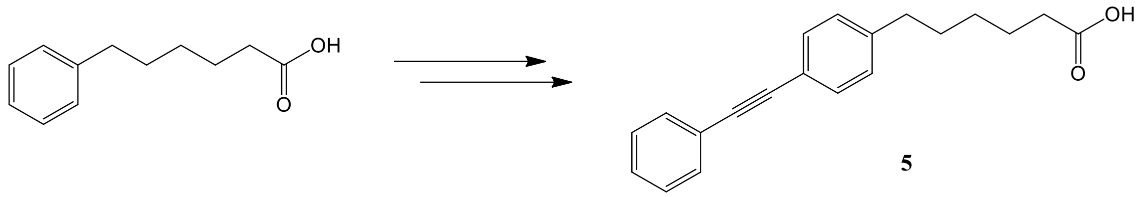

5

Product 5 was synthesized from 6-phenylhexanoic acid (1.0g, 5.20 mmol) employing the same procedure used for product 4. However, its iodo-derivative at the first step could not be recrystallized because of its oily nature. Therefore, crude products was used in each step and finally, the crude product at the last step was recrystallized from hexane-chloroform to yield product 5 (226mg, 15% over 4 steps); ^{1}H-NMR (400 MHz, $CDCl_3$, δ): 7.53-7.51 (m, 2H), 7.45 (d, 2H), 7.36-7.32 (m, 3H), 7.15 (d, 2H), 2.63 (t, 2H), 2.36 (t, 2H), 1.66 (q, 4H), 1.39 (m, 2H); ESI HRMS calcd for $C_{20}H_{20}O_2$ $[M+Na]^+$: 315.1361, found 315.1353.

Ethyl 2-(4-bromophenethoxy)acetate (6a)

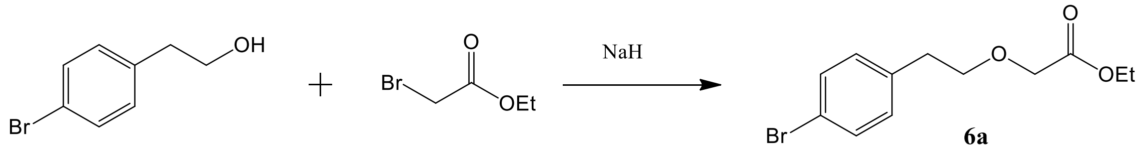

6a

Sodium hydride (60% in mineral oil 220 mg, 5.47 mmol)) was suspended in 15 ml of THF at 0°C. 2-(4-Bromophenyl)ethan-1-ol (1.0 g, 4.97 mmol) were added gradually and stirred for 30 minutes at 0 °C. Ethyl 2-bromoacetate (0.5 mL, 4.48 mmol) was added dropwise to the solution and stirred at room temperature for two hours. 20 ml of water was added to the solution then extracted three times with ethyl acetate. The combined organic layers were washed with brine and then dried over magnesium sulfate. The solvent was evaporated under reduced pressure and the residue was purified by column chromatography (hexane : ethyl acetate=10:1(v/v)) to yield the product (785mg, 55%); ^{1}H-NMR (400 MHz, $CDCl_3$, δ) : 7.35 (d, 2H), 7.09 (d, 2H), 4.15 (t, 2H), 4.02 (s, 2H), 3.69 (t, 2H), 2.84 (t, 2H), 1.22 (t, 3H).

Ethyl 2-(4-(phenylethynyl)phenethoxy)acetate (6b)

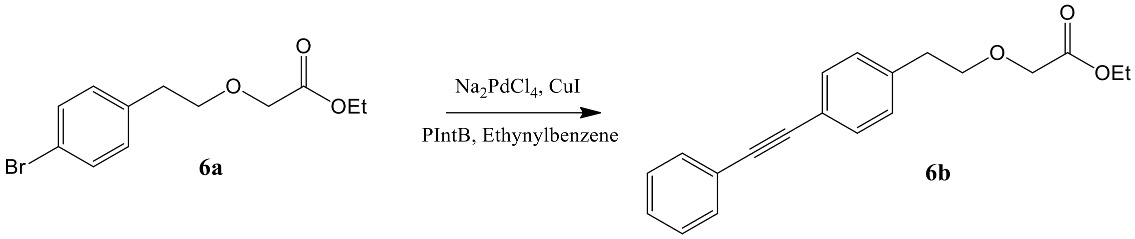

Ethyl 2-(4-bromophenethoxy)acetate (**6a**) (785 mg, 2.73 mmol) was dissolved in 5 ml of N, N, N', N'- tetramethylethylendiamine (TMEDA) and 0.5 ml of H_2O. Disodium tetrachloropalladate (Na_2PdCl_4, 7.3 mg, 0.02 mmol), Copper (I) iodide (9.5 mg, 0.05 mmol), 2-(di-*tert*-butylphosphino)-N-phenylindole (PIntB, 20 mg, 0.05 mmol), ethynylbenzene (0.27 mL, 2.49 mmol) was added and the reaction was stirred at 80 °C under Ar until all starting material was consumed, as was monitored by TLC. Water was added to the solution then extracted three times with ethyl acetate. The combined organic layers were washed two times with saturated ammonium chloride, brine and then dried over magnesium sulfate. The solvent was evaporated under reduced pressure and the residue was purified by column chromatography (hexane : ethyl acetate=4:1(v/v)) to yield the product (676 mg, 80%); ^{1}H-NMR (400 MHz, $CDCl_3$, δ) : 7.52-7.45 (m, 4H), 7.30-7.18 (m, 5H), 4.15 (q, 2H), 4.02 (s, 2H), 3.69 (t, 2H), 2.84 (t, 2H), 1.22 (t, 3H).

2-(4-(Phenylethynyl)phenethoxy)acetic acid (6)

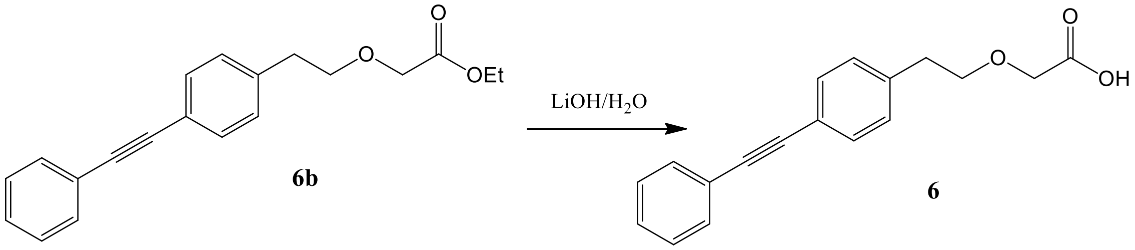

Ethyl 2-(4-(phenylethynyl)phenethoxy)acetate (**6b**) (676 mg, 2.19 mmol) was dissolved in 8 mL of THF, and lithium hydroxide monohydrate (185 mg, 4.38 mmol) dissolved in 4 ml of H_2O was added to the solution and stirred at room temperature until all the starting material was consumed, as was monitored by TLC. Water was added to the solution and the pH was adjusted to pH < 1 with 3% HCl solution. The combined organic layers were extracted three times with ethyl acetate, washed with brine, and then dried over magnesium sulfate. The solvent was evaporated under reduced pressure to yield the product **6** (583 mg, 95%); ^{1}H-NMR (400 MHz, $CDCl_3$, δ): 7.53-7.50 (m, 2H), 7.46 (d, 2H), 7.35-7.31 (m, 3H), 7.21 (d, 2H), 4.12 (s, 2H), 3.77 (t, 2H), 2.95 (t, 2H); ESI HRMS calcd for $C_{18}H_{16}O_3$ $[M+Na]^+$: 303.0992, found 303.0990.

Methyl 3-((4-bromobenzyl)oxy)propanoate (7a)

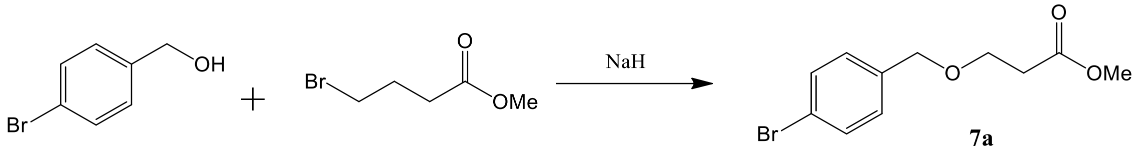

4-Bromophenyl methanol (1.0 g, 5.35 mmol) was suspended in 15 ml of toluene. Sodium hydride (60% in mineral oil 320 mg, 8.02 mmol) were added gradually and stirred for 30 min. Methyl 4-bromobutanoate (0.7mL, 6.42mmol) was added drop wise to the solution and stirred overnight at 50 °C. 20 ml of H_2O was added to the solution then extracted three times with ethyl acetate. The combined organic layers were washed with brine and then dried over magnesium sulfate. The solvent was evaporated under reduced pressure and the residue was purified by column chromatography (hexane : ethyl acetate=10:1(v/v)) to yield the product (920mg, 63%); ^{1}H-NMR (400 MHz, $CDCl_3$, δ): 7.47 (d, 2H), 7.25 (d, 2H), 4.55 (s, 2H), 3.76 (t, 2H), 3.67 (s, 3H), 2.68 (t, 2H).

Methyl 3-((4-(phenylethynyl)benzyl)oxy)propanoate (7b)

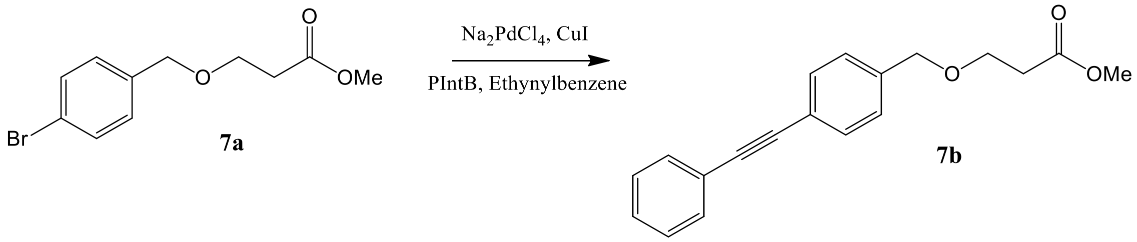

Methyl 3-((4-bromobenzyl)oxy)propanoate (**7a**) (920 mg, 3.38 mmol) was dissolved in 6 ml of N, N, N’, N’- tetramethylethylendiamine (TMEDA) and 1 ml of H_2O. Disodium tetrachloropalladate (Na_2PdCl_4, 9 mg, 0.03 mmol), Copper (I) iodide (12 mg, 0.06 mmol), 2-(di-*tert*-butylphosphino)-N-phenylindole (PIntB, 27 mg, 0.06 mmol), ethynylbenzene (0.34 mL, 3.07 mmol) was added and the reaction was stirred at 80 °C under Ar until all starting material was consumed, as was monitored by TLC. Water was added to the solution then extracted three times with ethyl acetate. The combined organic layers were washed two times with saturated ammonium chloride, brine and then dried over magnesium sulfate. The solvent was evaporated under reduced pressure and the residue was purified by column chromatography (hexane : ethyl acetate=4:1(v/v)) to yield the product (978 mg, 98%); ^{1}H-NMR (400 MHz, $CDCl_3$, δ): 7.54-7.50 (m, 4H), 7.35-7.30 (m, 5H), 4.55 (s, 2H), 3.76 (t, 2H), 3.67 (s, 3H), 2.68 (t, 2H).

3-((4-(Phenylethynyl)benzyl)oxy)propanoic acid (7)

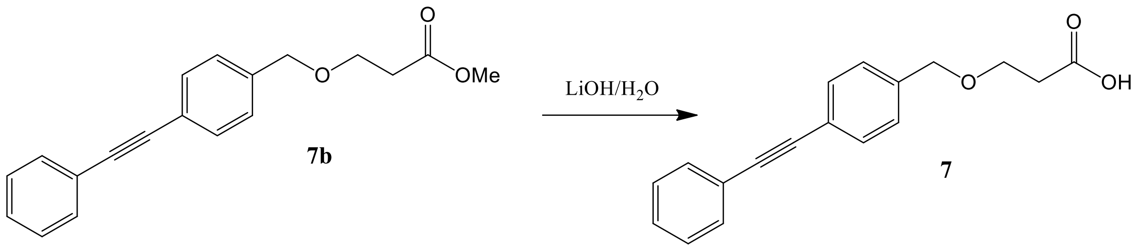

Methyl 3-((4-(phenylethynyl)benzyl)oxy)propanoate (**7b**) (978 mg, 3.32 mmol) was dissolved in 15 mL of THF, and lithium hydroxide monohydrate (280 mg, 6.65 mmol) dissolved in 7 ml of H_2O was added to the solution and stirred at room temperature until all the starting material was consumed, as was monitored by TLC. Water was added to the solution and the pH was adjusted to pH < 1 with 3% HCl solution. The combined organic layers were extracted three times with ethyl acetate, washed with brine, and then dried over magnesium sulfate. The solvent was evaporated under reduced pressure to yield the product **7** (700 mg, 75%); ^{1}H-NMR (400 MHz, $CDCl_3$, δ): 7.54-7.50 (m, 4H), 7.35-7.30 (m, 5H), 4.55 (s, 2H), 3.76 (t, 2H), 2.68 (t, 2H); ESI HRMS calcd for $C_{18}H_{16}O_3$ $[M+Na]^+$: 303.0992, found 303.0986.

Ethyl 2-(4-(naphthalen-2-ylethynyl)phenethoxy)acetate (9a)

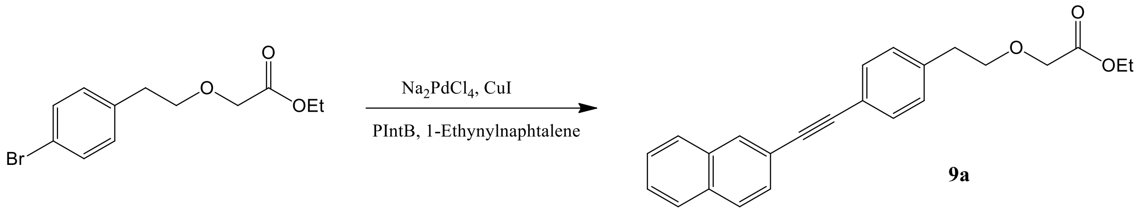

Ethyl 2-(4-bromophenethoxy)acetate (110 mg, 0.38 mmol) was dissolved in 1 ml of N, N, N', N'- tetramethylethylendiamine (TMEDA) and 0.1 ml of H_2O. Disodium tetrachloropalladate (Na_2PdCl_4, 1.1 mg, 3.48 umol), Copper (I) iodide (1.5 mg, 6.96 umol), 2-(di-*tert*-butylphosphino)-N-phenylindole (PIntB, 3.0 mg, 6.97 umol), 2-ethynylnaphthalene (53 mg, 0.35 mmol) was added and the reaction was stirred at 80 °C under Ar until all starting material was consumed, as was monitored by TLC. Water was added to the solution then extracted three times with ethyl acetate. The combined organic layers were washed two times with saturated ammonium chloride, brine and then dried over magnesium sulfate. The solvent was evaporated under reduced pressure and the residue was purified by column chromatography (hexane : ethyl acetate=8:1(v/v)) to yield the product (72 mg, 58%); ^{1}H-NMR (400 MHz, $CDCl_3$, δ): 8.05 (s, 1H), 7.83-7.80 (m, 3H), 7.59-7.49 (m, 5H), 7.24 (d, 2H), 4.23 (q, 2H), 4.12 (s, 2H), 3.83 (t, 2H), 2.99 (t, 2H), 1.22 (t, 3H).

2-(4-(Naphthalen-2-ylethynyl)phenethoxy)acetic acid (9)

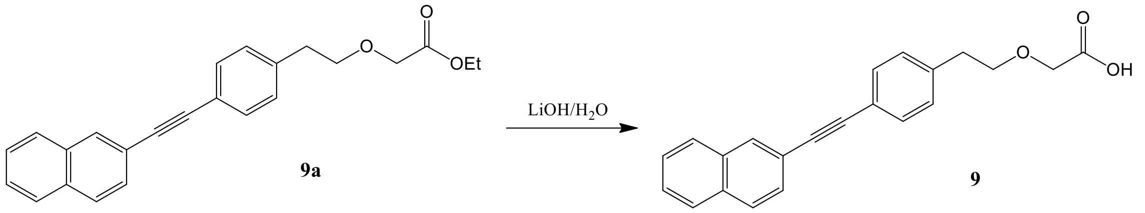

Ethyl 2-(4-(naphthalen-2-ylethynyl)phenethoxy)acetate (**9a**) (72 mg, 0.20 mmol) was dissolved in 1.5 mL of THF, and lithium hydroxide monohydrate (17 mg, 0.40 mmol) dissolved in 0.5 ml H2O was added to the solution and stirred at room temperature until all the starting material was consumed, as was monitored by TLC. Water was added to the solution and the pH was adjusted to pH < 1 with 3% HCl solution. The combined organic layers were extracted three times with ethyl acetate, washed with brine, and then dried over magnesium sulfate. The solvent was evaporated under reduced pressure to yield the product 9 (62 mg, 93%); 1H-NMR (400 MHz, CDCl3, δ): 8.05 (s, 1H), 7.83-7.80 (m, 3H), 7.59-7.49 (m, 5H), 7.24 (d, 2H), 4.12 (s, 2H), 3.83 (t, 2H), 2.99 (t, 2H). ESI HRMS calcd for $C_{22}H_{18}O_3$ $[M+Na]^+$:353.1154, found 353.1150.

Ethyl 2-(4-(pyren-1-ylethynyl)phenethoxy)acetate (10a)

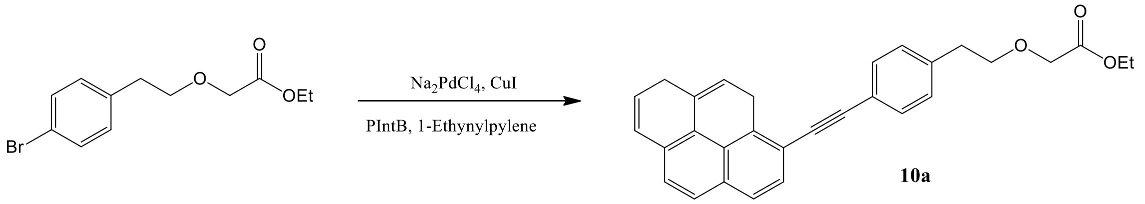

Ethyl 2-(4-bromophenethoxy)acetate (100 mg, 0.35 mmol) was dissolved in 1 ml of N, N, N’, N’- tetramethylethylendiamine (TMEDA) and 0.1 ml of H_2O. Disodium tetrachloropalladate (Na_2PdCl_4, 1.0 mg, 3.17 umol), Copper (I) iodide (1.2 mg, 6.33 umol), 2-(di-*tert*-butylphosphino)-N-phenylindole (PIntB, 2.5 mg, 6.33 umol), 1-ethynylpyrene (72 mg, 0.32 mmol) was added and the reaction was stirred at 80 °C under Ar until all starting material was consumed, as was monitored by TLC. Water was added to the solution then extracted three times with ethyl acetate. The combined organic layers were washed two times with saturated ammonium chloride, brine and then dried over magnesium sulfate. The solvent was evaporated under reduced pressure and the residue was purified by column chromatography (hexane : ethyl acetate=8:1(v/v)) to yield the product (70 mg, 51%); ^{1}H-NMR (400 MHz, $CDCl_3$, δ): 8.67 (d, 1H), 8.23-8.04 (m, 8H), 7.68 (d, 2H), 7.31 (d, 2H), 4.23 (q, 2H), 4.11 (s, 2H), 3.82 (t, 2H), 3.02 (t, 2H), 1.30 (t, 3H).

2-(4-(Pyren-1-ylethynyl)phenethoxy)acetic acid (10)

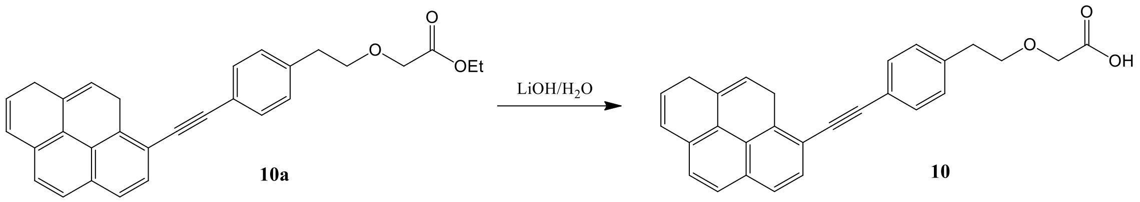

Ethyl 2-(4-(pyren-1-ylethynyl)phenethoxy)acetate (**10a**) (70 mg, 0.16 mmol) was dissolved in 1.5 mL of THF, and lithium hydroxide monohydrate (14 mg, 0.32 mmol) dissolved in 0.5 ml of H_2O was added to the solution and stirred at room temperature until all the starting material was consumed, as was monitored by TLC. Water was added to the solution and the pH was adjusted to pH < 1 with 3% HCl solution. The combined organic layers were extracted three times with ethyl acetate, washed with brine, and then dried over magnesium sulfate. The solvent was evaporated under reduced pressure to yield the product **10** (64 mg, 98%); ^{1}H-NMR (400 MHz, $CDCl_3$, δ): 8.67 (d, 1H), 8.23-8.04 (m, 8H), 7.68 (d, 2H), 7.31 (d, 2 H), 4.15 (s, 2H), 3.87 (t, 2H), 3.02 (t, 2H). ESI HRMS calcd for $C_{28}H_{22}O_3$ $[M+Na]^+$: 429.1467, found 429.1470

Ethyl 2-(4-((4-methoxyphenyl)ethynyl)phenethoxy)acetate (11a)

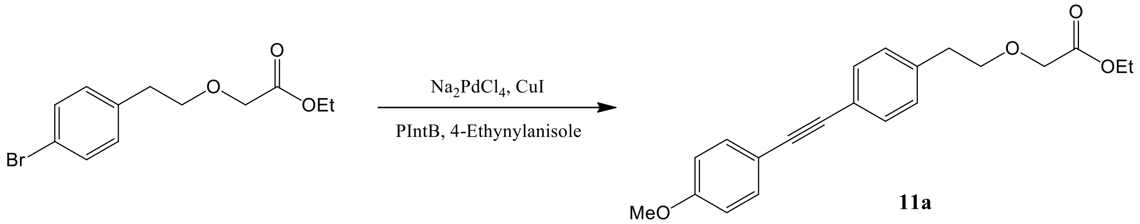

Ethyl 2-(4-bromophenethoxy)acetate (100 mg, 0.35 mmol) was dissolved in 1 ml of N, N, N’, N’- tetramethylethylendiamine (TMEDA) and 0.1 ml of H_2O. Disodium tetrachloropalladate (Na_2PdCl_4, 1.0 mg, 3.17 umol), Copper (I) iodide (1.2 mg, 6.33 umol), 2-(di-*tert*-butylphosphino)-N-phenylindole (PIntB, 2.5 mg, 6.33 umol), 4-ethynylanisole (41 uL, 0.32 mmol) was added and the reaction was stirred at 80 °C under Ar until all starting material was consumed, as was monitored by TLC. Water was added to the solution then extracted three times with ethyl acetate. The combined organic layers were washed two times with saturated ammonium chloride, brine and then dried over magnesium sulfate. The solvent was evaporated under reduced pressure and the residue was purified by column chromatography (hexane : ethyl acetate=8:1(v/v)) to yield the product (35 mg, 35%); ^{1}H-NMR (400 MHz, $CDCl_3$, δ: 7.45 (tt, 4H), 7.22 (d, 2H), 6.88 (d t, 2H), 4.22 (q, 2H), 4.08 (s, 2H), 3.83 (s, 3H), 3.76 (t, 2H), 2.96 (t, 2H), 1.29 (t, 3H).

2-(4-((4-Methoxyphenyl)ethynyl)phenethoxy)acetic acid (11)

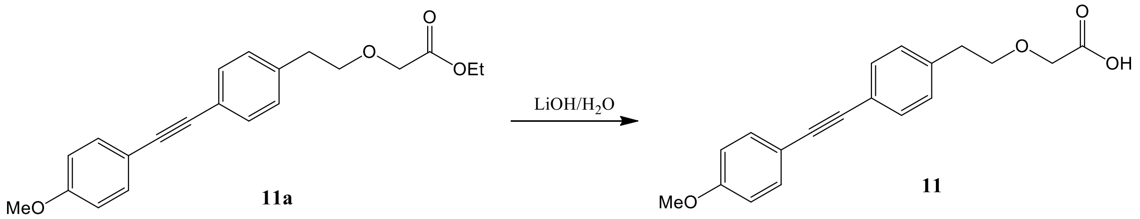

Ethyl 2-(4-((4-methoxyphenyl)ethynyl)phenethoxy) acetate (**11a**) (35 mg, 0.10 mmol) was dissolved in 1.0 mL of THF, and lithium hydroxide monohydrate (9 mg, 0.21 mmol) dissolved in 0.5 ml of H_2O was added to the solution and stirred at room temperature until all the starting material was consumed, as was monitored by TLC. Water was added to the solution and the pH was adjusted to pH < 1 with 3% HCl solution. The combined organic layers were extracted three times with ethyl acetate, washed with brine, and then dried over magnesium sulfate. The solvent was evaporated under reduced pressure to yield the product **11** (31 mg, 97%); ^{1}H-NMR (400 MHz, $CDCl_3$, δ): 7.45 (tt, 4H), 7.22 (d, 2H), 6.88 (d t, 2H), 4.08 (s, 2H), 3.83 (s, 3H), 3.76 (t, 2H), 2.96 (t, 2H). ESI HRMS calcd for $C_{19}H_{18}O_4$ $[M+Na]^+$: 333.1103, found 333.1100.

Ethyl 2-(4-((4-cyanophenyl)ethynyl)phenethoxy)acetate (12a)

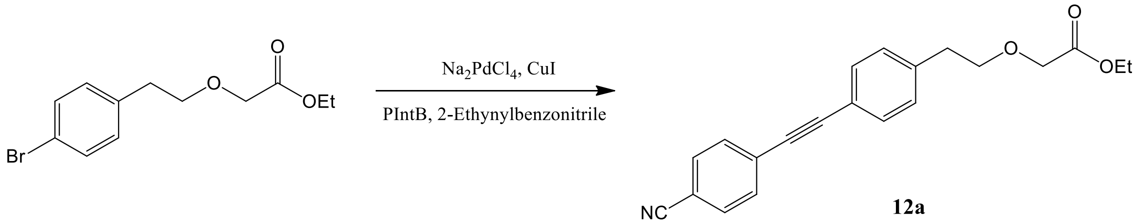

Ethyl 2-(4-bromophenethoxy) acetate (112 mg, 0.39 mmol) was dissolved in 2 mL of N, N, N’, N’- tetramethylethylendiamine (TMEDA) and 0.5 ml of H_2O. Disodium tetrachloropalladate (Na_2PdCl_4, 1.1 mg, 3.90 umol), Copper (I) iodide (1.5 mg, 7.80 umol), 2-(di-*tert*-butylphosphino)-N-phenylindole (PIntB, 3.11 mg, 7.80 umol), 4-Ethynylbenonitrile (149 mg, 1.17 mmol) was added and the reaction was stirred at 80 °C under Ar until all starting material was consumed, as was monitored by TLC. Water was added to the solution then extracted three times with ethyl acetate. The combined organic layers were washed two times with saturated ammonium chloride, brine and then dried over magnesium sulfate. The solvent was evaporated under reduced pressure and the residue was purified by column chromatography (hexane : ethyl acetate=8:1(v/v)) to yield the product (37 mg, 29%); ^{1}H-NMR (400 MHz, $CDCl_3$, δ): 7.64-7.58 (m, 4H), 7.47 (d, 2H), 7.27 (d, 2H), 4.22 (q, 2H), 4.08 (s, 2H), 3.78 (t, 2H), 2.98 (t, 2H), 1.28 (t, 3H).

2-(4-((4-cyanophenyl)ethynyl)phenethoxy)acetic acid (12)

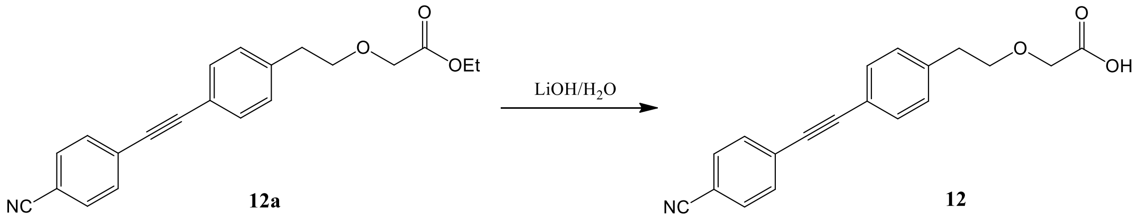

12a 12

Ethyl 2-(4-((4-cyanophenyl)ethynyl)phenethoxy)acetate (**12a**) (35 mg, 0.11 mmol) was dissolved in 2.0 mL of THF, and lithium hydroxide monohydrate (9 mg, 0.21 mmol) dissolved in 0.5 ml of H_2O was added to the solution and stirred at room temperature until all the starting material was consumed, as was monitored by TLC. Water was added to the solution and the pH was adjusted to pH < 1 with 3% HCl solution. The combined organic layers were extracted three times with ethyl acetate, washed with brine, and then dried over magnesium sulfate. The solvent was evaporated under reduced pressure to yield the product **12** (18 mg, 56%); ^{1}H-NMR (400 MHz, $CDCl_3$, δ): 7.65-7.58 (m, 4H), 7.49 (d, 2H), 7.25 (d, 2H), 4.11 (s, 2H), 3.82 (t, 2H), 2.98 (t, 2H). ESI HRMS calcd for $C_{19}H_{15}NO_3$ $[M+Na]^+$: 328.0950, found 328.0945.

HPLC profiles and molecular weight of of PNAs

PNA0 K-ATCTCCTCCCTT

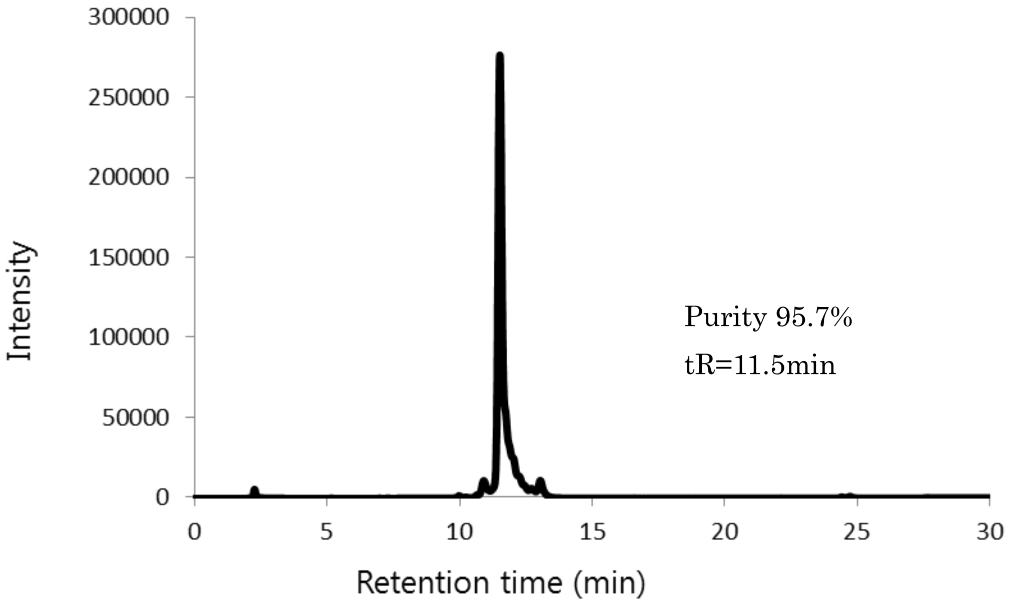

M.W. Calcd: 3258.38, found: 3261.74

PNA1 K-ATCTCCTCCCTT-**1**

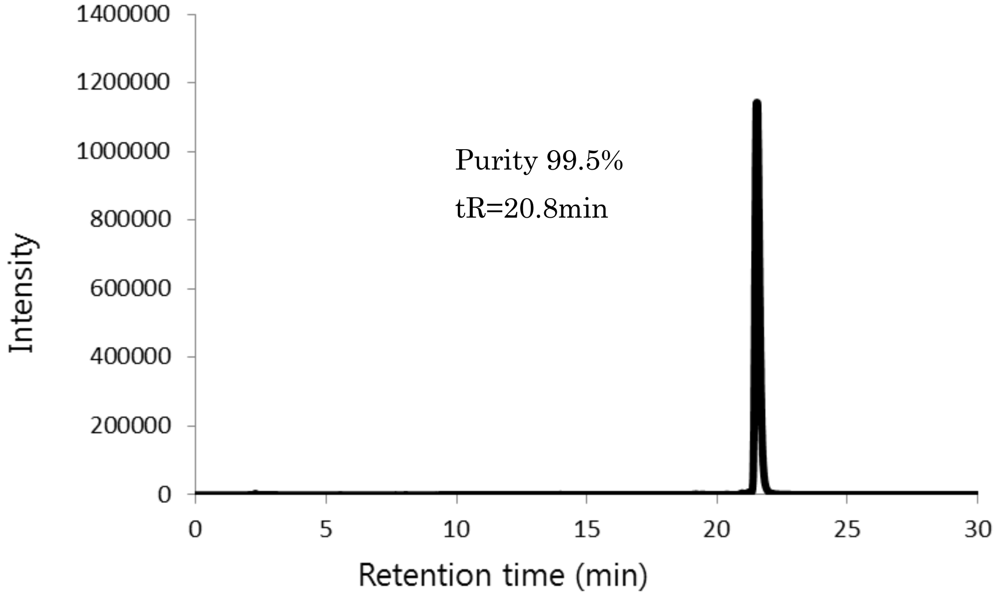

M.W. Calcd: 3490.67, found: 3495.84

PNA2 K-ATCTCCTCCCTT-**2**

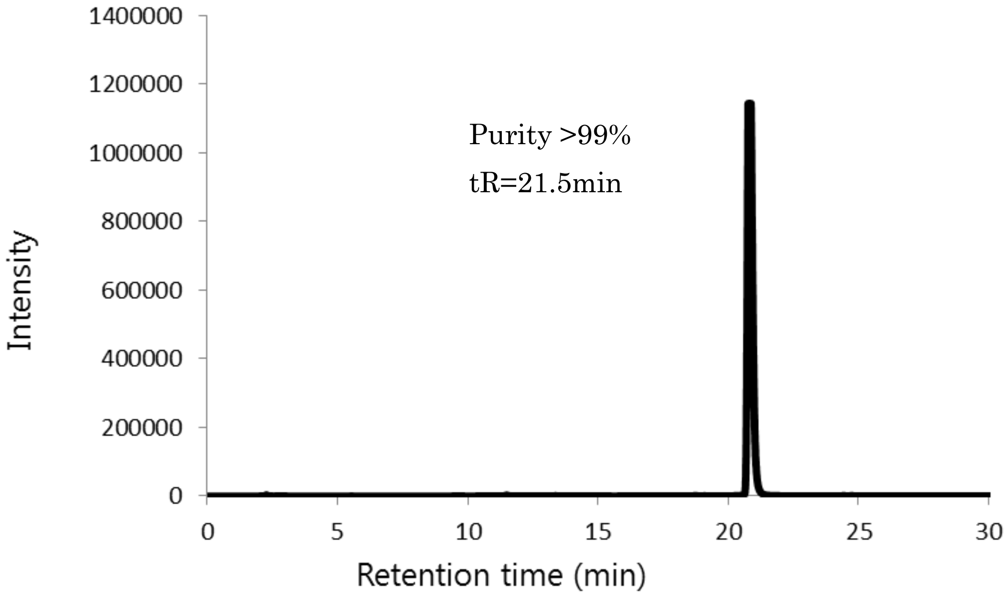

M.W. Calcd: 3476.65, found: 3482.33

PNA3 K-ATCTCCTCCCTT-3

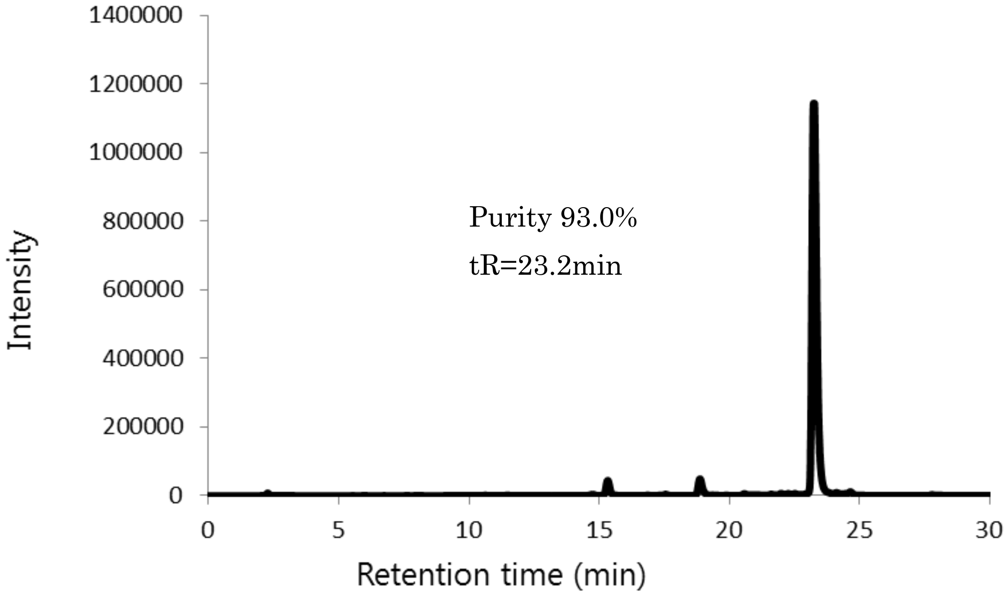

M.W. Calcd: 3504.70, found: 3508.71

PNA4 K-ATCTCCTCCCTT-4

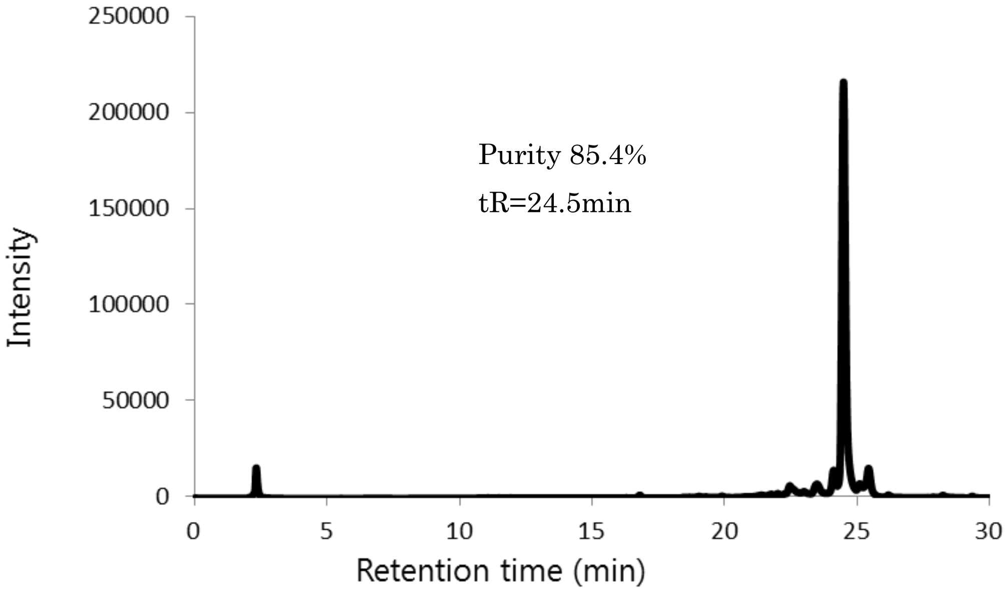

M.W. Calcd: 3518.73, found: 3519.78

PNA5 K-ATCTCCTCCCTT-**5**

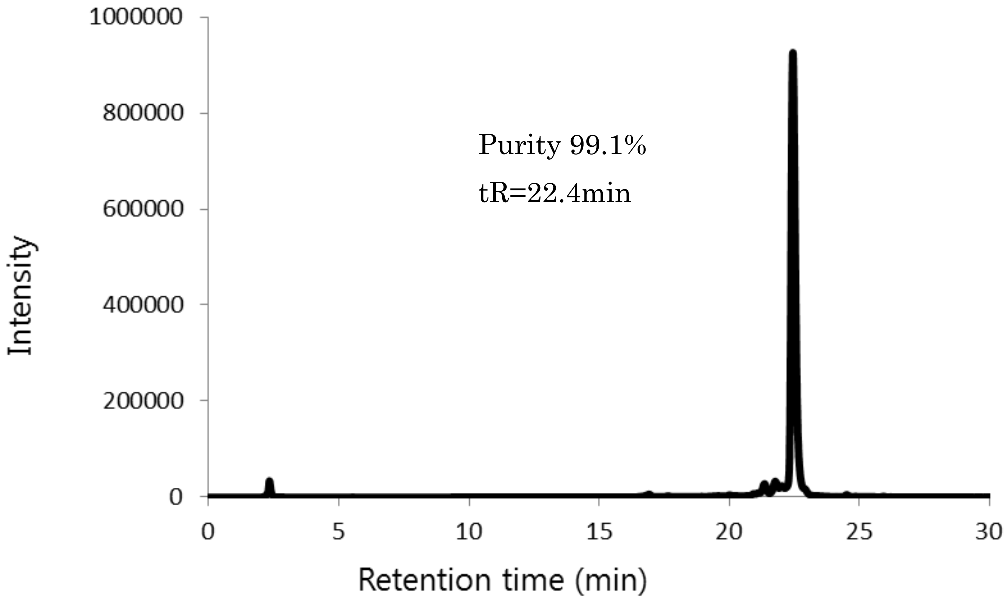

M.W. Calcd: 3532.76, found: 3536.00

PNA6 K-ATCTCCTCCCTT-**6**

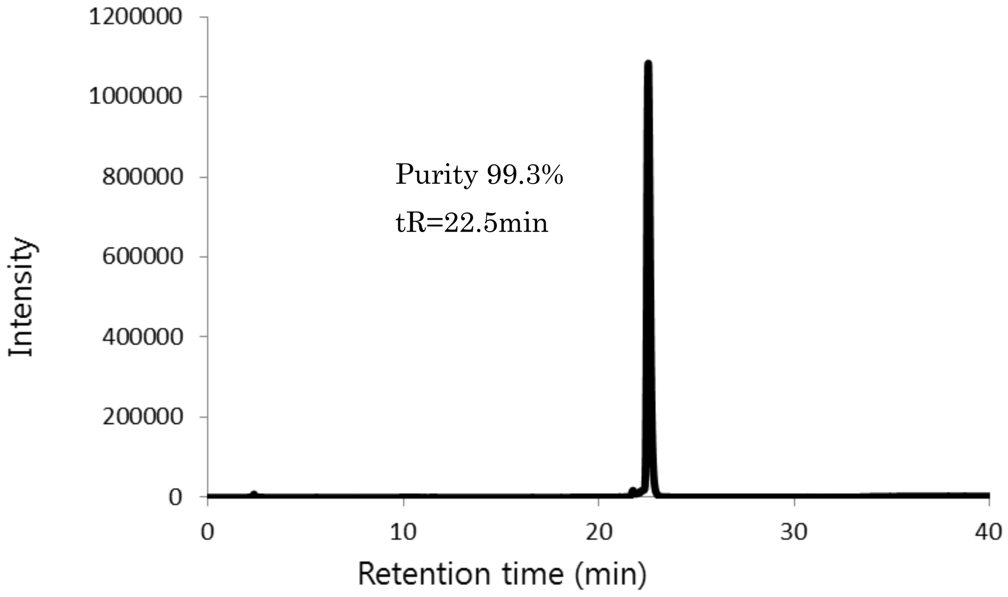

M.W. Calcd: 3520.70, found: 3520.79

PNA7 K-ATCTCCTCCCTT-**7**

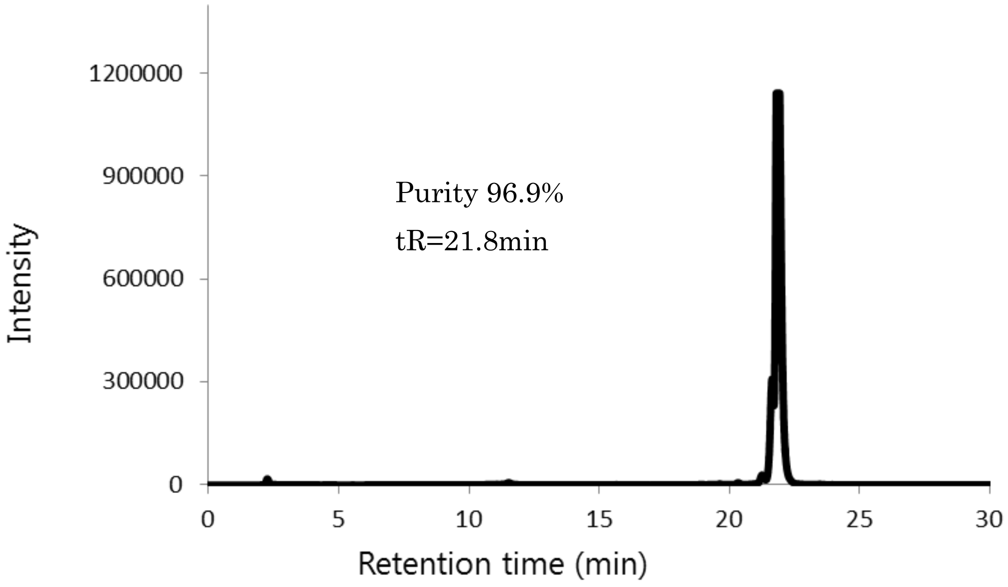

M.W. Calcd: 3520.70, found: 3521.16

PNA8 K-ATCTCCTCCCTT-Gly-**2**

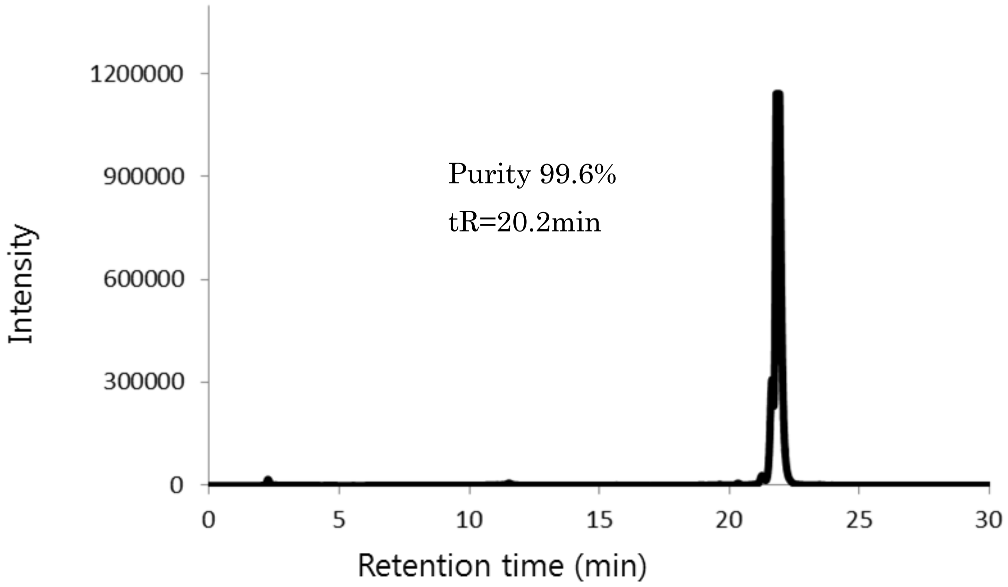

M.W. Calcd: 3533.72, found: 3534.27

PNA9 K-ATCTCCTCCCTT-**9**

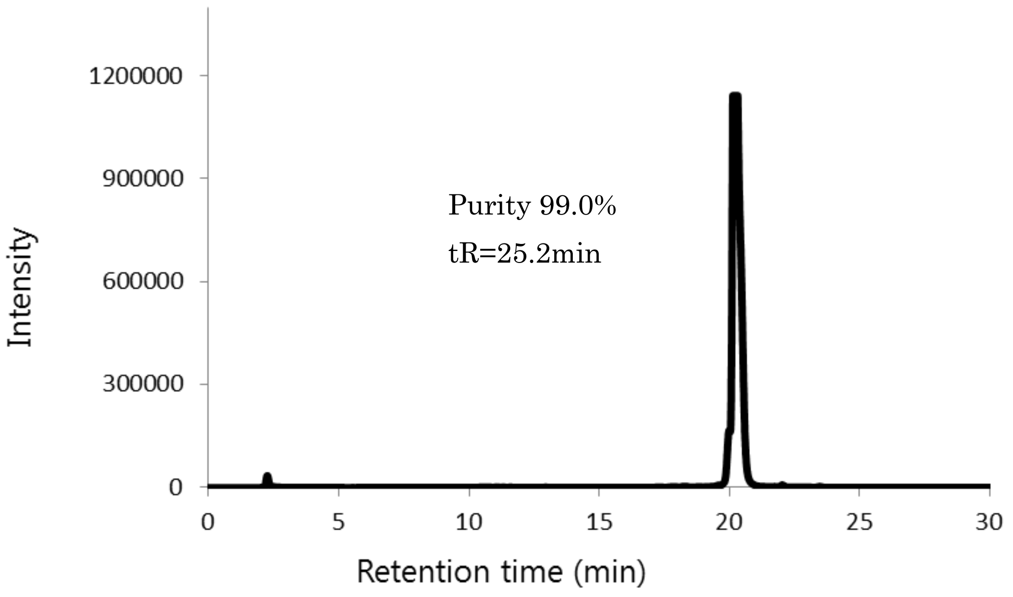

M.W. Calcd: 3570.76, found: 3570.68

PNA10 K-ATCTCCTCCCTT-**10**

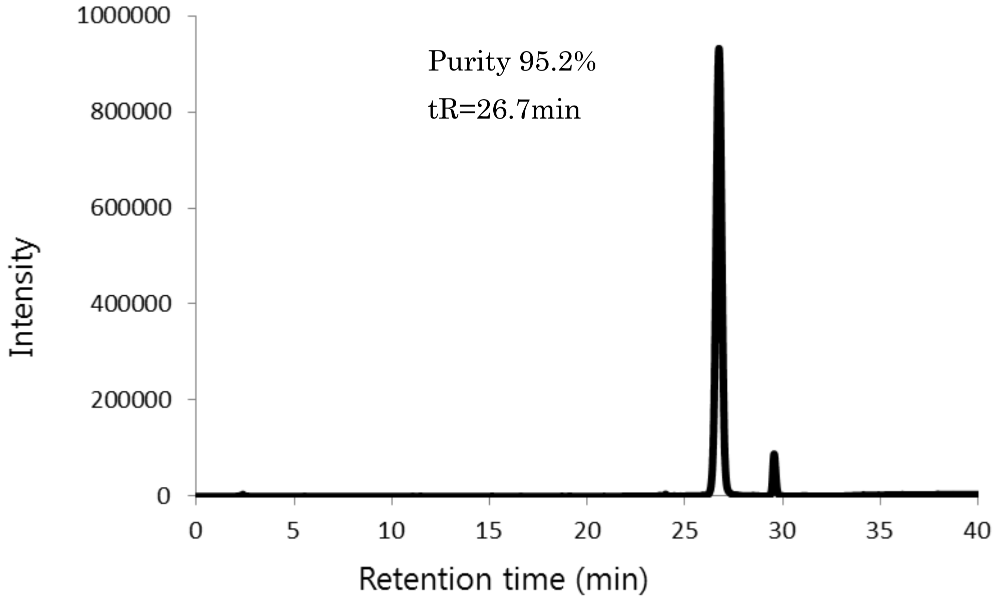

M.W. Calcd: 3644.85, found: 3642.05

PNA11 K-ATCTCCTCCCTT-**11**

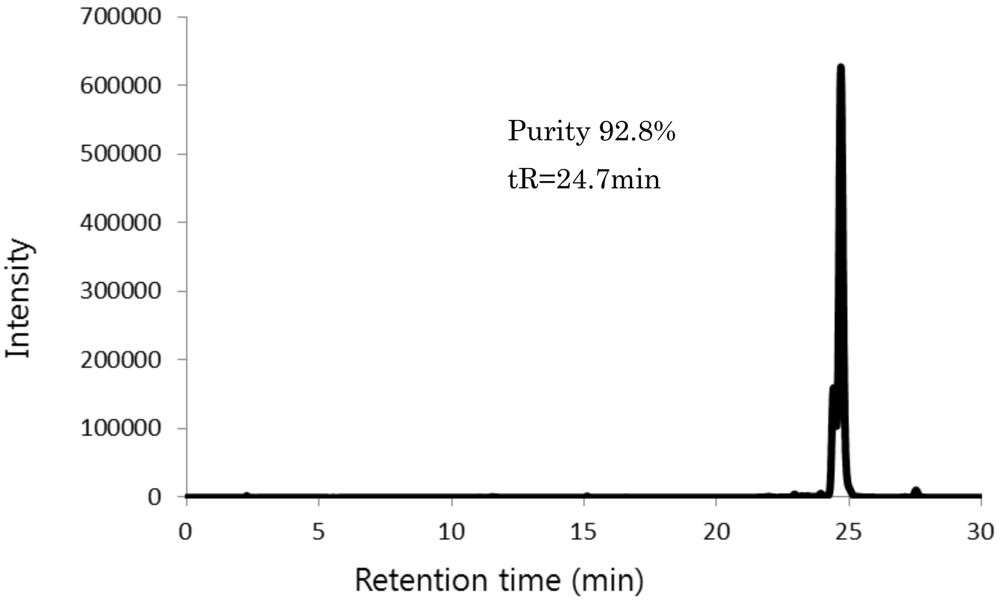

M.W. Calcd: 3550.73, found: 3552.13

PNA12 K-ATCTCCTCCCTT-**12**

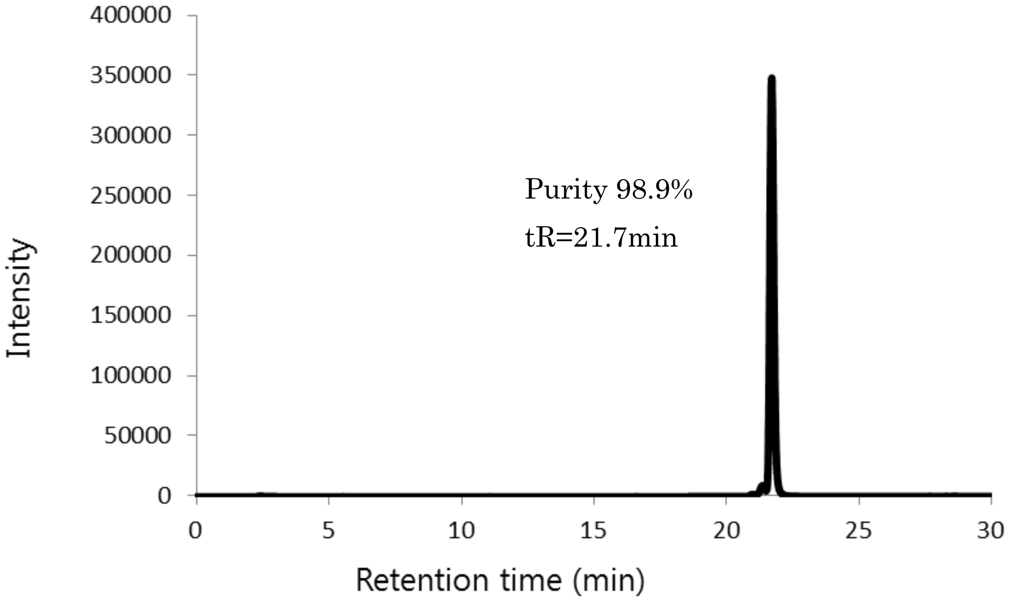

M.W. Calcd: 3545.71, found: 3546.78

PNA13 K-ATCTCCTCCCTT-13

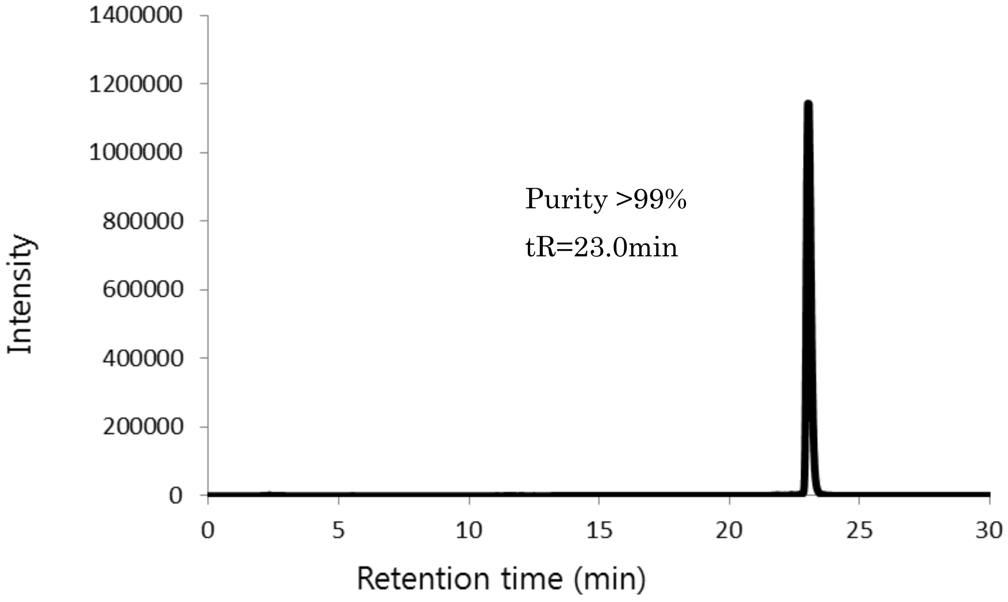

M.W. Calcd: 3538.69, found: 3539.03

Abbreviations

O: 2-aminoethoxy-2-ethoxy acetic acid

BCIP: 5-bromo-4-chloro-3'-indolylphosphatase p-toluidine salt

Bhoc: benzhydryloxycarbonyl

Boc: tert-butyloxycarbonyl

cDNA: complementary DNA

DMF: N,N-dimethylformamide

Fmoc: 9-fluorenylmethoxycarbonyl

HBTU: 2-(1H-benzotriazole-1-yl)-1,1,3,3-tetramethyluronium hexafluorophosphate

HOBt: N-hydroxybenzotriazole

NMM: N-methylmorpholine

TFA: trifluoroacetic acid

PNA: peptide nucleic acid

Reference

1. Christiansen, E.; Due-Hansen, M. E.; Urban, C.; Grundmann, M.; Schmidt, J.; Hansen, S. V.; Hudson, B. D.; Zaibi, M.; Markussen, S. B.; Hagesaether, E.; Milligan, G.; Cawthorne, M. A.; Kostenis, E.; Kassack, M. U.; Ulven, T. Discovery of a potent and selective free fatty acid receptor 1 agonist with low lipophilicity and high oral bioavailability. *J. Med. Chem.* **2013,** 56, 982-992.
